# Exploring cyclic networks of multisite modification reveals origins of information processing characteristics

**DOI:** 10.1038/s41598-020-73045-9

**Published:** 2020-10-06

**Authors:** Thapanar Suwanmajo, Vaidhiswaran Ramesh, J. Krishnan

**Affiliations:** 1 Center of Excellence in Materials Science and Technology, Chiang Mai University, Chiang Mai, 50200 Thailand; 2 Department of Chemistry, Faculty of Science, Chiang Mai University, Chiang Mai, 50200 Thailand; 3 Department of Chemical Engineering, Centre for Process Systems Engineering, Imperial College London, London, SW7 2AZ UK; 4 Institute for Systems and Synthetic Biology, Imperial College London, South Kensington Campus, London, SW7 2AZ UK

**Keywords:** Computational biology and bioinformatics, Systems biology

## Abstract

Multisite phosphorylation (and generally multisite modification) is a basic way of encoding substrate function and circuits/networks of post-translational modifications (PTM) are ubiquitous in cell signalling. The information processing characteristics of PTM systems are a focal point of broad interest. The ordering of modifications is a key aspect of multisite modification, and a broad synthesis of the impact of ordering of modifications is still missing. We focus on a basic class of multisite modification circuits: the cyclic mechanism, which corresponds to the same ordering of phosphorylation and dephosphorylation, and examine multiple variants involving common/separate kinases and common/separate phosphatases. This is of interest both because it is encountered in concrete cellular contexts, and because it serves as a bridge between ordered (sequential) mechanisms (representing one type of ordering) and random mechanisms (which have no ordering). We show that bistability and biphasic dose response curves of the maximally modified phosphoform are ruled out for basic structural reasons independent of parameters, while oscillations can result with even just one shared enzyme. We then examine the effect of relaxing some basic assumptions about the ordering of modification. We show computationally and analytically how bistability, biphasic responses and oscillations can be generated by minimal augmentations to the cyclic mechanism even when these augmentations involved reactions operating in the unsaturated limit. All in all, using this approach we demonstrate (1) how the cyclic mechanism (with single augmentations) represents a modification circuit using minimal ingredients (in terms of shared enzymes and sequestration of enzymes) to generate bistability and oscillations, when compared to other mechanisms, (2) new design principles for rationally designing PTM systems for a variety of behaviour, (3) a basis and a necessary step for understanding the origins and robustness of behaviour observed in basic multisite modification systems.

## Introduction

Multisite phosphorylation wherein substrates are modified at multiple sites by kinases/phosphatases is fundamental to cellular biology. It represents a basic way of encoding substrate function in cellular systems, and is encountered in a wide variety of cellular contexts. The interest in multisite phosphorylation stems from the convergence of two distinct aspects: as a mechanism for conferring substrate identity and function on one hand, and as a complex biochemical information processor on the other. Multisite phosphorylation (and more generally multisite modification) represent basic circuits/networks of post-translational modification (PTM), which are encountered in many cellular contexts, and consequently understanding their information processing characteristics is of broad relevance and interest.

Multisite phosphorylation is a recurrent feature of cell signalling networks^1–5^, while serving to encode substrate function^6–8^. It has important roles in a range of contexts such as the cell cycle regulatory system, Alzheimer’s disease and inflammation^8–12^. A key focal point involves multisite modification where the kinases and/or the phosphatases are the same. An examination of double-site modification with a common kinase and phosphatase reveals variations along different axes. One axis is the ordering of modifications: the modification may be ordered (sometimes called sequential) or random depending on whether or not a specific order of modification is imposed. Another axis of classification stems from the modification mechanism itself: if the enzyme is released after every modification, it is called distributive, whereas if the enzyme remains bound it is referred to as processive. Combinations of both mechanisms may also be observed. Depending on combinations of the above features (modification network “topology” and enzymatic mechanism) and the commonality of kinases and/or phosphatases, multiple basic modification scenarios can be isolated even in the double site modification system. With an increase in number of modification sites, many more variations are possible.

Elucidating the dynamics and information processing characteristics of post-translational modification networks and circuits, presents many challenges, and is the focus of a number of studies (see^13^ for a recent survey). It is worth pointing out that basic chemical and physical factors along with sequestration of species are key ingredients responsible for the complexity of the resulting behaviour. There are many studies focussing on different aspects of information processing in multisite phosphorylation. The capability of exhibiting threshold-like behaviour even with similar catalytic constants for multiple modifications by an enzyme was shown in^14^. The surprising feature that bistability could be seen in double site modification was shown in^15^ and this was generalized to reveal an unlimited multistability as the number of modification sites was increased^16^. A range of related systems have been studied in this context^17^. The fact that simple ordered distributive mechanism contained an in-built trade-off creating the possibility of biphasic responses was demonstrated in^18^. Oscillatory behaviour has been revealed in different cases: in random mechanisms with a single kinase/phosphatase pair, and also resulting from the interplay of distributive and processive mechanisms in ordered modification systems, or even arising from an enzyme activation step^19–21^. Other studies have focussed on the presence or absence of oscillations in these systems^22,23^. Processive systems by contrast behave in a simpler way and exhibit a unique asymptotically stable steady state^24–26^. In a recent paper we examined multiple transitions in intrinsic behaviour which emerged as a consequence of modification mechanisms being part of pathways^21^. Other studies have studied a range of related aspects^27–33^.

**Figure 1:**
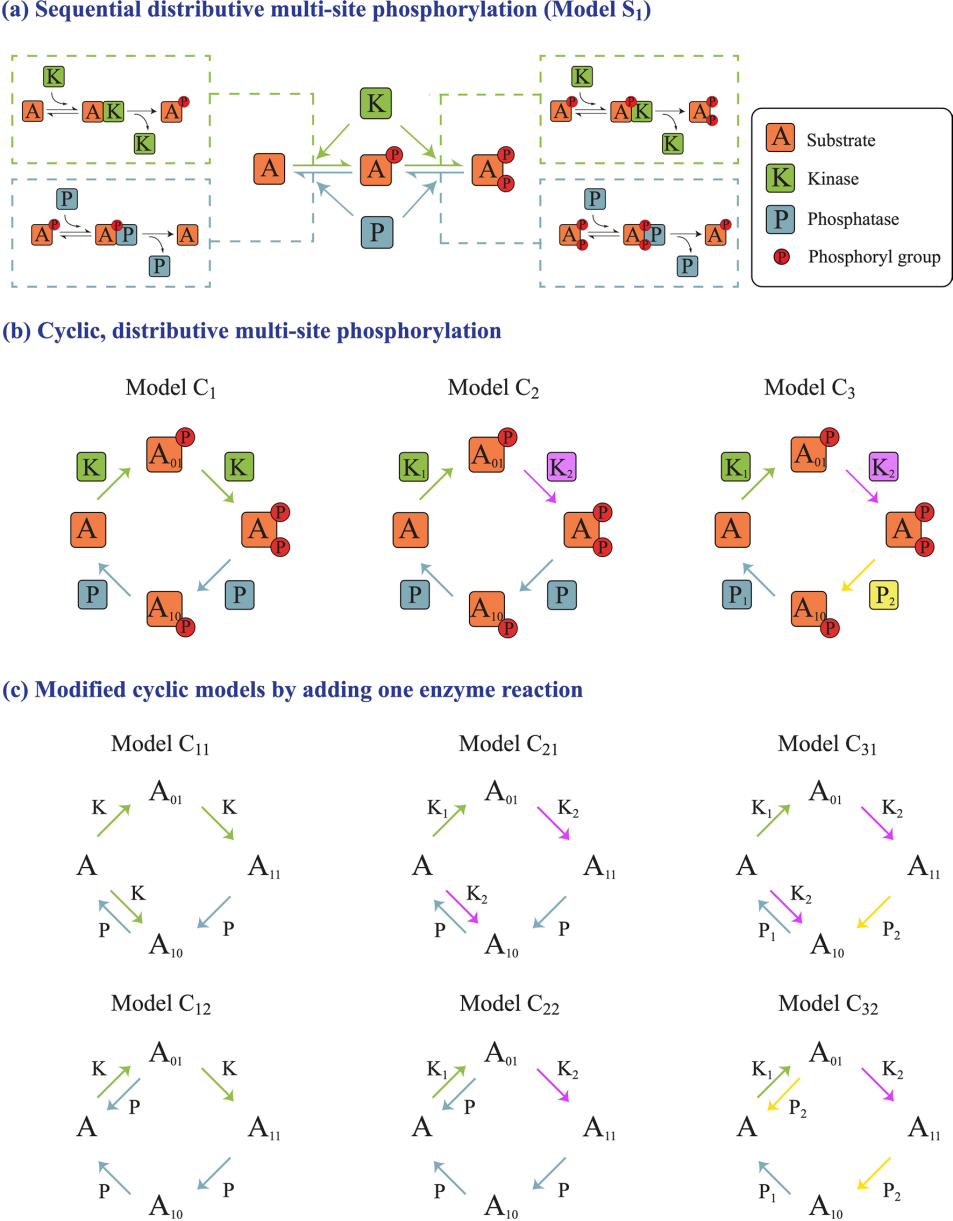
Schematic of multi-site phosphorylation models. (**a**) Sequential distributive (double-site) phosphorylation mechanisms illustrating the basic steps. (**b**) Cyclic distributive double-site phosphorylation models with single kinase and phosphatase (model $C_1$), multiple kinases and single phosphatase (model $C_2$) and multiple kinases and phosphatases (model $C_3$). (**c**) Modified cyclic models (described in (**b**)) with an extra enzymatic reaction. Models $C_{11}$, $C_{21}$, $C_{31}$ involve an extra kinase-mediated step, while models $C_{12}$, $C_{22}$ and $C_{32}$ involve an extra phosphatase-mediated step.

A fundamental ingredient of multisite phosphorylation is the ordering of the modifications. We focus in this paper on a variant of multisite phosphorylation, corresponding to a basic ordering of the modifications, which has received little attention: the cyclic mechanism. To illustrate this we focus on the double site modification. When the order of phosphorylation and dephosphorylation are opposite, then we obtain the much studied ordered sequential mechanism. This is characterized by a single partial phosphoform analogues, which is a direct consequence of the ordering. There is however another basic ordered mechanism, one where the order of phosphorylation and dephosphorylation are the same (Fig. 1). This ordering results in two partial phosphoforms. This system combines specific features of ordered and random mechanisms: a specific ordering on one hand, and a network topology akin to random mechanisms on the other. We employ a structured systems approach to analyze different facets of the cyclic mechanism. We examine multiple variants of the basic cyclic mechanism with common/separate kinases and common/separate phosphatases, noting that the commonality or difference of modifying enzymes is a fundamental aspect of multisite modification. We then address the question as to what minimal augmentations in the cyclic mechanism (relaxing the strict requirement of ordering of modifications) can give rise to behaviour seen in random mechanisms. Our focus is on key characteristic information processing: bistability, oscillatory behaviour and biphasic dose responses in the maximally modified phosphoforms. These have been extensively studied in other models, and are of natural interest in signalling pathways. We then comment on analogues of cyclic mechanisms when there are a greater number of modifications.

There are multiple reasons which justify the focus and approach of the study. Firstly cyclic mechanisms represent a very basic variant of multisite phosphorylation (based on a specific ordering of modifications). Thus they represent a basic model PTM system. Secondly, cyclic multisite phosphorylation/dephosphorylation are seen in multiple natural cellular contexts. One example is rhodopsin phosphorylation, which is responsible for dark adaptation^34,35^. This has an extra step of arrestin binding which maintains the cyclic structure. Mutations in rhodopsin kinase and arrestin are implicated in Oguchi’s disease^36,37^. Another example relevant to bacterial clocks, is the post-translational oscillator involving Kai proteins (with a cyclic structure), embedded in a transcription–translation feedback loop^38^ (also see^39^ which has a similar structure, but with some extra steps). Thirdly, there are other ways in which a circuit very similar to the cyclic network can arise: this can happen if for instance the phosphorylation involves substrate binding to a scaffold, while dephophorylation occurs without binding to a scaffold. If an intermediate phosphoform is not released from the scaffold during phosphorylation, then we have a mechanism which is structurally essentially the same as the cyclic mechanism (note that this ensures that the phosphorylation and dephosphorylation act on different partial phosphoform analogues), with minor augmentations. Another way in which a similar modification logic as the cyclic mechanism can be generated is phosphorylation and dephosphorylation occur in different compartments, with only unmodified and fully modified forms shuttling (eg. see^40^). In our paper, we examine relaxations of the basic cyclic structure, to create a bridge between ordered and random mechanisms. This line of investigation may be relevant in multiple contexts which involve random mechanisms with some modification steps irreversible. In particular, the ERK signalling network involves phosphorylation at two sites, with phosphorylation (mediated by MEK) proceeding in the same order as dephosphorylation (mediated by MKP3)^41^. This ordering corresponds to a cyclic network, and different studies have considered other additional reactions built on to this^15,30,42^. Finally, our study serves as a basis for engineering chemical modification in synthetic biology to achieve specific information processing tasks^43,44^.

By examining cyclic models with different variants with separate/common kinases and phosphatases, we can obtain basic design insights into which combinations of basic ingredients enable or prevent certain types of behaviour. By systematically augmenting these circuits, we can identify the minimum augmentations (and the specific nature of the augmentations of the circuit) to enable different kinds of behaviour. Through this approach we can also study the relationship between the cyclic mechanism, the ordered sequential mechanism and the random mechanism, recognizing that the cyclic mechanism is a bridge between those well-studied mechanisms. In this way, we are able to elucidate and synthesize the impact of ordering of modifications of these systems on the overall behaviour. All in all, we are also able to draw a sharp boundary between the impossibility and possibility of different types of behaviour, and distill the interplay between reaction topology, commonality and sequestration of enzymes which enables different behaviour, through our approach. In addition to the relevance to systems and synthetic biology, the study also has relevance to the broader field of chemical information processing, which aims to dissect and engineer different patterns of information processing in chemical systems, with applications as diverse as analytical chemistry and chemical computation.

## Models and methods

A (sequential) ordered mechanism (henceforth also referred to as sequential or ordered) of double site modification where the order of phosphorylation is exactly opposite to the order of dephosphorylation is a mechanism which results in only one partial phosphoform (Fig. 1a). The random mechanism of double site modification has two intermediate phosphoforms, and reflects the fact that phosphorylation and dephosphorylation can proceed in any order. A cyclic mechanism represents specific ordering of phosphorylation and dephosphorylation: in double site modification it corresponds to the situation where the ordering of dephosphorylation is the same as that of phosphorylation (see Fig. 1b).

We use a double-site modification with a cyclic mechanism, as a basic focal point throughout the paper. We also briefly examine, cyclic mechanisms involving more than two site modifications. The basic cyclic mechanism (Fig. 1b) involves the unphosphorylated form (denoted $A_{00}$ or just *A*) modified by a kinase in sequence to give $A_{01}$ and $A_{11}$: this represents an ordering where one of the sites (whose modification status is represented by the second subscript) is modified first, after which the other site is modified. However since the site modified by the kinase first is also the first to be demodified by the phosphatase, this gives rise to the intermediate phosphoform $A_{10}$ which is further modified to give $A_{00}$. This corresponds to a strict ordering of phosphorylation and dephosphorylation. Implicit in this model is the fact that phosphorylation and dephosphorylation occur primarily in specific sequences, and residual levels of any other phosphorylation or dephosphorylation is neglected. In this regard we note that if these other steps were included, when the residual levels of these other phosphorylation/dephosphorylation steps are sufficiently weak (for fixed total amounts of species) the essential conclusions remain the same. We also examine cases where some of these assumptions regarding ordering are relaxed (see below).

The model of the cyclic modification network is built up as follows. All models of individual modification steps (phosphorylation, dephosphorylation) are described through widely-used models involving the relevant enzyme binding to substrate to form the complex which irreversibly modifies the substrate to the modified form (see Fig. S1 for a detailed schematic representation of the reactions): this assumes an excess of ATP. When a single kinase and single phosphatase act to perform all the modifications, we assume a distributive modification, wherein the enzyme unbinds from the substrate after effecting a single modification.

We examine multiple variants of cyclic mechanisms, based on the identities of the enzymes involved in multiple modifications, whether common or distinct (Fig. 1b). Thus we examine the common kinase-common phosphatase mechanism, the different kinase-common phosphatase mechanism as well as the different kinase-different phosphatase mechanism. These variants are all relevant noting that (1) there are many instances of enzymes performing multiple modifications of a substrate (justifying the common kinase-common phosphatase variant) (2) kinases outnumber phosphatases are seen in genome-wide studies^45^ (justifying the different kinase, common phosphatase variant).

We also examine specific relaxations of the assumptions of the cyclic mechanisms (see Fig. 1c). For instance, we examine the case that one of the enzymes (either kinase or phosphatase) may not be subject to a specific ordering. This has the consequence of creating an extra reaction in the cyclic reaction network and we examine the consequence of this as well. The networks depicted in Fig. 1c consider the possibility that the extra reaction may be mediated by a kinase or a phosphatase. Additional cases of an added extra reaction to the cyclic network are considered and discussed in “Supplementary material”. We point out that the essential kinds of insights we draw are already seen in the networks in Fig. 1c.

The models for all these variations are built up in the same way, implementing a mass action description for the kinetics of the individual modification steps. The mass action description does not make a-priori assumptions regarding regimes of enzyme activity and thus offers a comphrehensive model of the original system. These models (all kinetic models) are presented in the “Supplementary material”. We defer the study of both stochastic and spatial aspects to a future study, once some basic aspects of modification kinetics are understood, and many of these aspects are most transparently understood in the ODE setting.

### Parameters and the focal point of the analysis

Our focal point is a range of specific qualitative behaviour (oscillations, multistability, biphasic responses of maximally modified phosphoforms) all of which are of experimental interest in natural or engineered biology. Our primary goal is to establish unambiguously, the presence or absence of these characteristics in the various networks considered. Thus our results involve a combination of computational results which demonstrate the possibility of such behaviour on one hand, along with analytical results which establish the absence of behaviour for structural reasons, independent of parameters. In the former case we briefly discuss the ranges of parameters which enable the behaviour to be seen. Through a combination of the two approaches, and by applying this to multiple variants of the cyclic multisite module, we are able to sharpen the boundary between the absence and presence of different types of behaviour. This directly reveals what modification network characteristics (structural and parametric) enables certain behavior to be seen and allows us to determine minimum requirements for a network to demonstrate the behaviour.

In our computational work, enzyme-substrate unbinding constants are fixed at the same value for all enzyme substrate pairs. This does not introduce any essential restriction and allows for the entire range of qualitative behaviour we focus on, to be seen in the models considered. The remaining parameters are the catalytic constants, the enzyme substrate binding constants, and total amounts of enzymes and substrate. We choose representative values for these parameters within plausible ranges studied in the literature, and study their effect if this results in the qualitative behaviour of interest. Our main computational goal is to establish the presence of different characteristic behaviour in the networks under consideration.

The models are simulated using the ode15s solver in MATLAB^46^. These MATLAB models were cross-validated with simulations of the same network in COPASI^47^. Note that the latter requires only specification of the network, parameters, and kinetics of individual steps. Bifurcation analysis of these models is performed using MATCONT^48^, after incorporating conservation conditions and eliminating certain variables. Computational studies are complemented by analytical approaches both on a given model, as well as variations (see Fig. 1), to pin down the origins of a particular observed behaviour.

## Results

### Basic cyclic networks

#### Steady state dose responses of the cyclic model.

**Figure 2:**
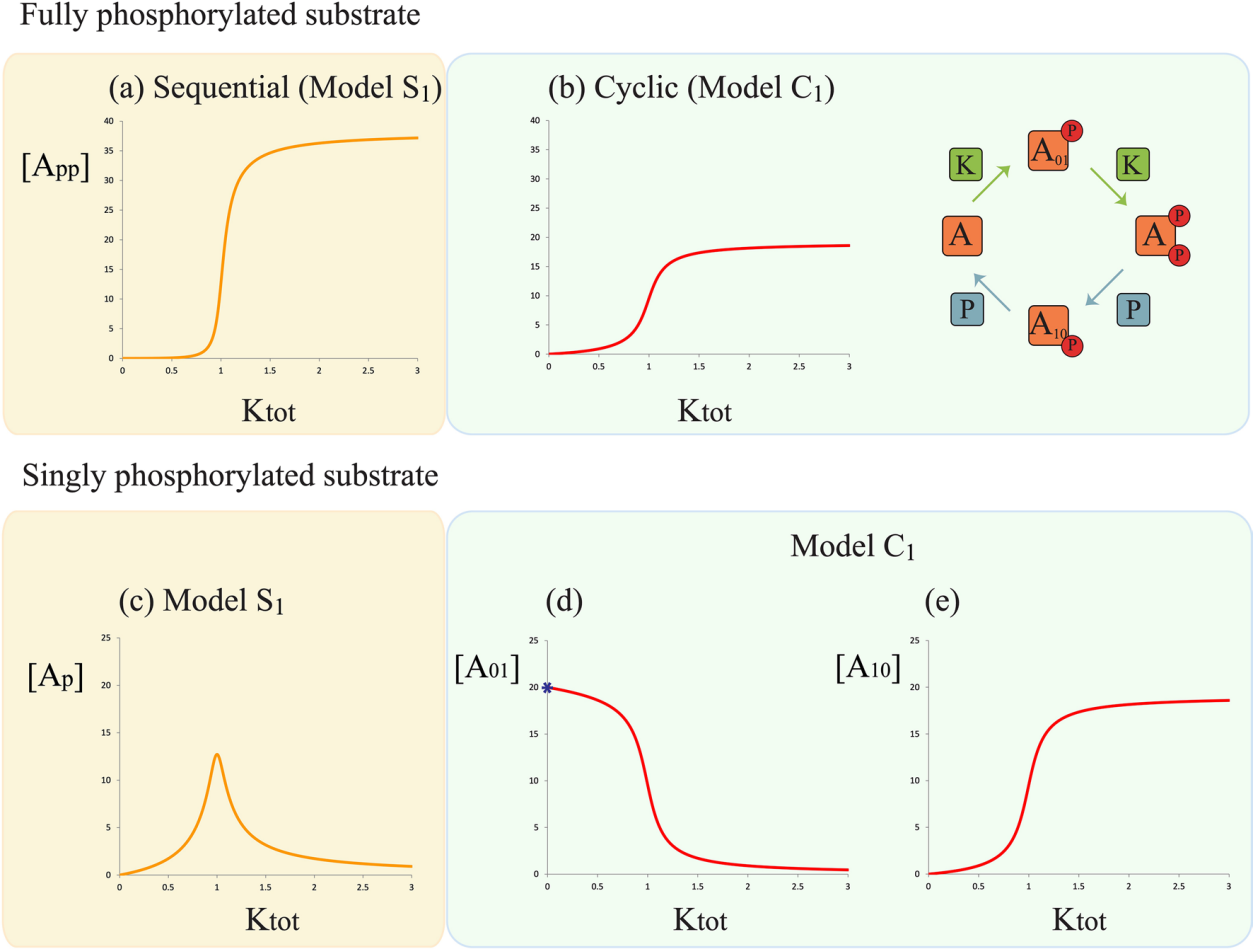
Steady-state dose–response curves in double site distributive phosphorylation with a single (common) kinase and a single (common) phosphatase: sequential distributive (model $S_1$) and cyclic mechanisms (model $C_1$). (**a**, **b**) Shows the steady-state input–output response curve of the concentration of the maximally phosphorylated substrate, $A_{pp}$, as a function of the total concentration of kinase, $K_{tot}$ in each of these models, while (**c**–**e**) shows the corresponding curve for the partially phosphorylated substrate, $A_p$ (in the sequential distributive model) and $A_{01}$ or $A_{10}$ (in the cyclic model).

We first examine the cyclic model with a single kinase and single phosphatase (Fig. 2). For purposes of contrast, we compare the behaviour of a cyclic mechanism with that of an ordered (sequential) distributive model. The enzyme binding/unbinding and catalytic constants associated with both the fully modified substrate, as well as the unmodified substrate (and associated enzymes) are exactly the same in the two models. Furthermore the enzyme/substrate binding, unbinding and catalytic constants for the relevant enzymes (kinase, phosphatase) and the partial phosphoform are exactly the same in the two models (note that the cyclic model has two partial phosphoforms). Figure 2a shows a monotonic dose response curve for the maximally modified phosphoform concentration as the total kinase amount is varied, in both the ordered sequential and the cyclic models, though the cyclic model has a quantitatively reduced output.

#### Behaviour of partial phosphoforms

A clear difference is seen in the case of partial phosphoforms. In the ordered distributive model, the partial phosphoform exhibits a characteristic biphasic response, which is associated with the fact that the kinase both produces and converts the partial phosphoform: for lower concentrations of kinase, the former behaviour dominates, while the latter behaviour is seen for higher total kinase concentrations. In the case of the cyclic model, the two partial phosphoforms exhibit monotonic responses, with opposite characteristics, one monotonically decreasing (which mirrors the unphosphorylated substrate $A_{00}$, also referred to below as *A*) and the other which is monotonically increasing (which mirrors the fully phosphorylated substrate $A_{11}$). The origins of this behaviour is worth discussing further.

#### Analysis of partial phosphoform behaviour

By examining the steady state of $A_{01}+A_{01}K$, we find that $[AK] \propto [A_pK]$. Furthermore, since the steady state concentration of a complex is proportional to the product of the enzyme and the substrate, we find that $[A_p] \propto [A]$ for any non-zero total kinase concentration. We note further that the cyclic model assumes no phosphatase acting on $A_{01}$. If a weak level of dephosphorylation is present in $A_{01}$, we find that when $K_{tot}$ becomes very small, then $[A_{01}]$ will actually approach zero. In this case, however, for relatively small $K_{tot}$, this partial phosphoform will increase and saturate following which with increasing $K_{tot}$, a monotonically decreasing profile as discussed is observed. This can be summarized by saying that the partial phosphoform behaves essentially like that seen above except for small levels of kinase, where any residual action of a phosphatase (neglected in this model) may dominate, making the concentration of the partial phosphoform approach zero. An analysis of the steady state of $A_{10}+A_{10}P$ yields the fact that $[A_{10}P] \propto [A_{11}P]$, from which it follows that $[A_{10}] \propto [A_{11}]$, which yields a characteristic increasing profile for $A_{10}$ mirroring that of $A_{11}$.

#### Absence of bistability

**Figure 3:**
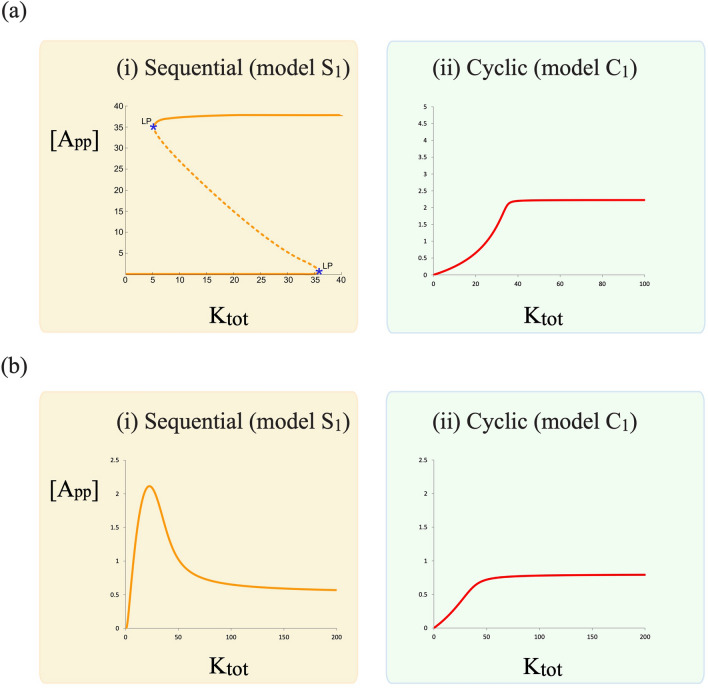
Steady-state dose–response curves in sequential distributive (model $S_1$) and cyclic phosphorylation (model $C_1$) models with different catalytic constants of phosphorylation reactions (see text). (**a**) Shows how bistability may be obtained in a sequential distributive model (1), while this behaviour is abolished in a cyclic model with a single kinase and single phosphatase (2). (**b**) Shows that the biphasic behaviour which can arise from a sequential distributive model (1) is not found in the cyclic model (2). [Dashed and thick lines represent unstable and stable steady states respectively, LP: denotes a limit point (saddle-node bifurcation)].

It is well known that the ordered (distributive) mechanism can result in bistability^14,15^ and analysis reveals the parameters which enable bistability^49^. In contrast, the cyclic mechanism for these parameter values results only in monostability (Fig. 3a). In fact a straightforward mathematical analysis (“Supplementary material”) reveals that the cyclic model will only produce a single steady state, irrespective of parameter values.

Another behaviour of interest is biphasic dose responses in the maximally modified phosphoform (e.g. in response to variation of total kinase concentrations), which have been shown both experimentally and theoretically. We had shown that the biphasic behaviour arises in the ordered distributive mechanism, because increasing kinase concentrations can sequester partial phosphoforms in complexes, thus allowing more phosphatase to target the fully modified form^18^.

#### Absence of biphasic dose-responses in the maximally modified phosphoform

For corresponding parameters (where an ordered model exhibits a biphasic dose response), the cyclic model only exhibits a monotonically increasing dose response curve for the fully modified phosphoform (Fig. 3b). Direct mathematical analysis shows that biphasic dose response curves are not possible in the basic model independent of parameters (see “Supplementary material”).

#### The existence of oscillations

**Figure 4:**
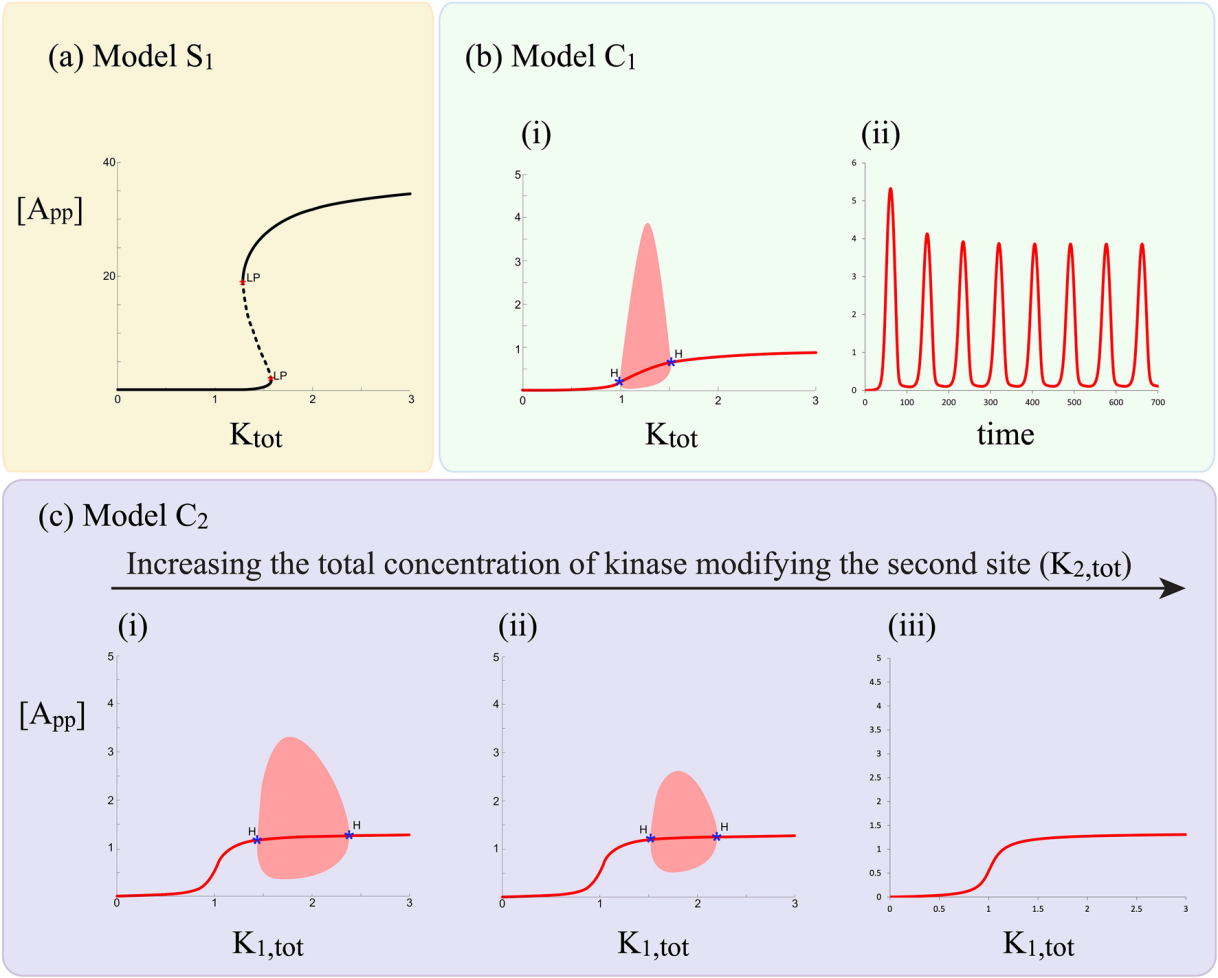
Oscillatory behaviour in cyclic double-site phosphorylation models. (**a**, **b**) Shows how bistability may be obtained in a sequential distributive model while the cyclic model with common kinase and common phosphatase (model $C_1$) is capable of generating sustained oscillations, for corresponding parameter values (see text). (**c**) Sustained oscillations can be found in the cyclic model with different kinases, and a common phosphatase (model $C_2$) in a range of total concentration of $K_{1,tot}$ and this behavior is progressively reduced and eventually destroyed by increasing the total concentration of the kinase modifying the second site ($K_{2,tot}$). [Dashed and thick lines represent unstable and stable steady states respectively; LP and H denote a limit point and Hopf bifurcation point respectively].

The previous results point to structural features of the cyclic model, which result in complex information processing characteristic of sequential (and random) distributive models being prevented, and suggests that the cyclic model behaves in a very simple way. This is in line with the intuition that the cyclic model which have “disconnected” forward and reverse modification pathways, might behave comparably to a single site modification system. However, Fig. 4 shows that the cyclic model can exhibit oscillations. The sequential distributive double-site modification model has not been shown to intrinsically exhibit oscillations. In this case, the incorporation of an activation step of a shared enzyme in the distributive modification is sufficient to generate oscillations, even if the reverse modification is mediated by different enzymes^21^, though oscillations in the intrinsic ordered distributive model have been elusive. The cyclic model does not require an explicit enzyme activation step to produce oscillations. Oscillations were found to occur in regimes where the sequential distributive model exhibited bistability, though this is neither necessary nor sufficient.

#### Oscillations, bistability and biphasic responses in other cyclic models

We then explored other aspects of the cyclic mechanism to isolate requirements for oscillations. We found (Fig. 4b) that a cyclic model with different kinases and a common phosphatase can indeed generate oscillations as well, though cyclic models with different kinases and phosphatases do not. Cyclic models with separate kinases and common or separate phosphatases will not exhibit either multistability or biphasic dose response curves for the maximally modified phosphoform (see “Supplementary material”).

#### Cyclic mechanisms and ordered mixed mechanisms

The behaviour of the cyclic model, preventing multistability, but generating oscillations is reminiscent of another mechanism we have studied, the mixed-mechanism^20^. The mixed mechanism for the ordered distributive double site modification has only one partial phosphoform, and this phosphoform is directly created and released only in one modification direction. The cyclic model has two different phosphoforms, and furthermore the structure allows for having common enzymes in one direction and separate enzymes in the other, generating oscillation, something which does not happen in the analogous mixed model. The mixed model has a processive step, associated with a common enzyme: if the opposing modification is mediated by separate enzymes, oscillations will not result as the system is similar to a three step irreversible reaction network with separate enzymes (two adding a phosphatase group each, and one completing reversing this in one step). In such a network there is no common enzyme shared between phosphoforms (while acting distributively). This indicates an essential extra flexibility inherent in the cyclic mechanism vis-a-vis the mixed mechanism.

### Augmentations to basic cyclic networks

We now examine the cyclic model as a bridge between ordered and random mechanisms of multisite modification. Random mechanisms with different combinations of different/common kinase and different/common phosphatase can all exhibit both multistability and oscillations^21^. The ordered (distributive) model exhibits bistability, but has not been shown to intrinsically exhibit oscillations (there are theoretical studies which rule out oscillations in certain regimes^23^). The basic cyclic model exhibits oscillations but not bistability or biphasic behaviour. We explored the minimum additions to the cyclic model to obtain bistability and biphasic dose response curves. This amounts to relaxing some restrictive assumptions regarding ordering in the basic cyclic model. To do this, we examined the augmentation of either a single phosphorylation step (opposite a given dephosphorylation step) or a single dephosphorylation step (opposite a phosphorylation step). This is done for three classes of cyclic models (common kinase/common phosphatase, different kinase/common phosphatase and different kinase/different phosphatase). This results in a series of six models (see Fig. 1c) which we examine (from the focal point of our study, the behaviour of other variants, can for the most part mapped on to one of these models, with regard to bistability and oscillations; other remaining cases can be analyzed in analogous terms: this is discussed in detail in “Supplementary material”). All these networks are characterized by having a cyclic network with an additional reaction, and essentially correspond to special cases of random mechanisms, where these steps are the dominant ones.

#### A single augmentation to the basic cyclic networks can enable realization of bistability

**Figure 5:**
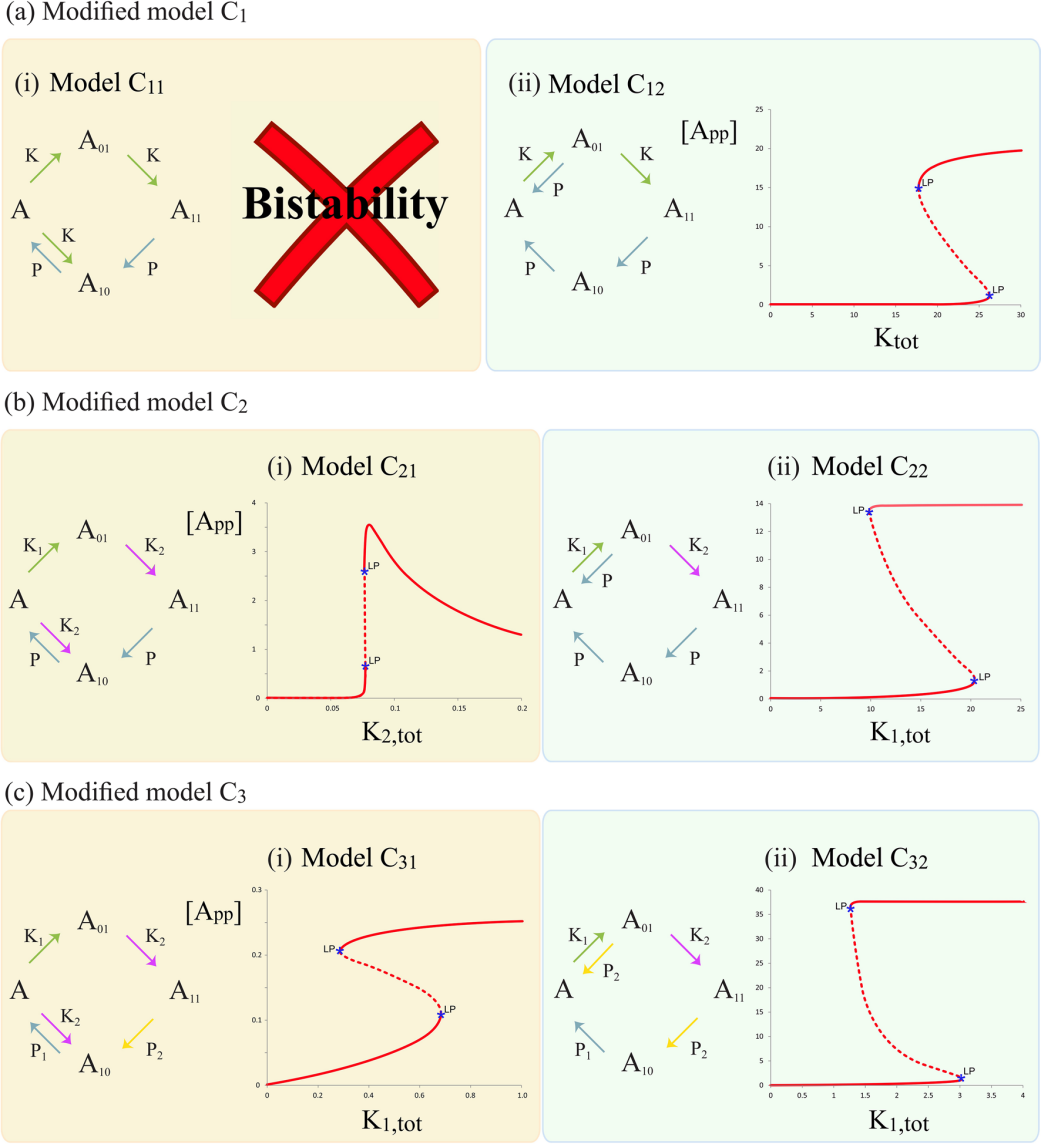
Bistability in modified cyclic models with a minimal augmentation of a single enzyme mediated step. (**a**) Indicates that the cyclic model with common kinase/phosphatase and an extra kinase reaction (model $C_{11}$) is incapable of generating bistability while the model with an extra phosphatase reaction (model $C_{12}$) can exhibit bistability. (**b**) Depicts cyclic models with different kinases/common phosphatase and an extra kinase-mediated (model $C_{21}$) or phosphatase-mediated augmentation (model $C_{22}$), both of which can exhibit bistability. (**c**) Shows the relevant cases for the different kinase/different phosphatase with kinase-mediated (model $C_{31}$) or phosphatase-mediated augmentation (model $C_{32}$), demonstrating that a single kinase or phosphatase mediated augmentation is sufficient for bistability. [Dashed and thick lines represent unstable and stable steady states respectively, LP: denotes a limit point].

Figure 5 focusses on the possibility of bistability. Figure 5a depicts one model with an extra phosphatase reaction and one model with an extra kinase reaction, in a cyclic mechanism with common kinase and common phosphatase. We see that the model C11 does not show bistability even with this augmentation of the cyclic mechanism, while model C12 readily exhibits bistability. Looking at both these cases carefully allows us to dissect the reasons for the different behaviour. Analytical work shows that in model C11, the new kinase reaction is still associated with the same substrate, and consequently the steady state equations have the same mathematical form as those of the basic cyclic mechanism (with altered parameters), for which bistability was precluded for structural reasons independent of parameters. On the other hand a single additional dephosphorylation in model C12 allows for bistability, because it results in a different enzyme substrate complex, which along with conservation is sufficient to generate bistability. These results also allows us to infer the behaviour of a different kinase reaction augmentation (acting on $A_{10}$) and a different phosphatase augmentation (acting on $A_{11}$). The former can give rise to bistability (it being similar in structure to model C12), while the latter does not exhibit bistability (for the same reasons as model C11).

Similar insights can be seen the Fig. 5b focussing on models with different kinases and a common phosphatase. A single kinase augmentation in model C21 gives rise to bistability, making an interesting contrast to model C11, since the extra augmentation is in the same reaction in the network. Here however, owing to the fact that this involves a different kinase, a new enzyme substrate complex is created and that along with conservation can give rise to bistability (along with a biphasic response, discussed later). Again a single phosphatase augmentation can give rise to bistability provided it acts on $A_{01}$ rather than $A_{11}$. Finally Fig. 5c shows the relevant cases for the different kinase and different phosphatase network showing that bistability owing to an augmentation can arise as long as new enzyme substrate complex is created. From analysing all the cases above, we see that such an augmentation is sufficient, irrespective of whether the kinases or phosphatases are common or separate.

#### The augmented reaction acting in the unsaturated regime is sufficient for creating bistability

We then examined whether the augmentation mentioned above can give rise to bistability, even if the additional reaction involved negligible sequestration (i.e. negligible complex, owing to the modification being in the unsaturated regime). In such cases the rate of substrate modification is linearly proportional to the relevant free enzyme and substrate concentration (this is obtained when the catalytic constant of modification becomes large). Computational analysis shows that in fact bistability can be obtained in all these cases (see Supplementary material, Fig. S2).

#### Oscillations in augmented cyclic networks

In the case of cyclic mechanisms with different kinases and phosphatases (where oscillations are not seen), having one additional reaction (see Model C31) can give rise to oscillations. Furthermore this is true even if this extra reaction is in the unsaturated limit (see “Supplementary material”).

#### Biphasic dose responses in augmented cyclic networks

Table 1 summarizes the cases for biphasic responses of the fully modified phosphoform as the total kinase concentration (and in the case of multiple kinases, each of the kinase concentrations) is varied. Model C11 precludes biphasic responses, irrespective of parameter values, as it has the same structure as the basic cyclic mechanism. Model C12 can readily show biphasic behaviour, since it demonstrates the key underpinning ingredients for this behaviour: kinase modifying intermediate phosphoforms, and sequestering them in complexes, making phophatase more easily available as a consequence. This was studied in^18^ and is seen in model C12, since the phosphatase targets $A_{01}$ (the phosphatase dephosphorylating $A_{10}$ is irrelevant for this).

#### The existence of robust biphasic responses

Model C21 exhibits biphasic response in relationship to both total kinase concentrations. Particularly noteworthy here is the response to variation in the second kinase (K2) concentration. Here we find a robust biphasic response for all other parameter values. Furthermore, as the total concentration of K2 is increased towards infinity, the $A_{11}$ concentration approaches zero. This is demonstrated analytically in “Supplementary material”. The biphasic response with respect to K1 is due to the fact that this reduces the production of $A_{10}$ through the K2 pathway (which has the effect of a reduced sequestration of phosphatase by this substrate, also see “Supplementary material”). Model C22 also exhibits biphasic response to K2 variation.

**Table 1:** A summary of biphasic behaviour for the fully modified substrate as the total kinase concentration is varied, in cyclic, distributive phosphorylation models with enzymatic augmentations (models C11, C12, C21, C22, C31, C32).

	Input	
	${K}_{{1,tot}}$ or ${K}_{{tot}}$	${K}_{{2,tot}}$
Model C31	No	Yes (robust)
Model C32	No	Yes$^{*}$
Model C21	Yes	Yes (robust)
Model C22	No	Yes$^{*}$
Model C11	No	
Model C12	Yes$^{*}$	

An asterisk indicates that the biphasic behavior is impossible if the augmentation is in the unsaturated regime (see text). Also indicated are cases where robust biphasic behavior is obtained.

#### Biphasic response to one kinase but not the other

Both models C31 and C32 can exhibit a biphasic response to variation of K2 but not to K1 (see “Supplementary material”). This absence of biphasic response to variation of K1 is demonstrated analytically for both cases. Again Model C31 exhibits a robust biphasic response to variation of total amount of K2.

#### Biphasic dose responses in cyclic networks, with the augmented reaction in the unsaturated regime

We probed the cases where biphasic responses could be obtained, by examining whether this remained true even if the augmented reaction was in the unsaturated regime (negligible sequestration). We found that the biphasic response to K2 in models C12, C22 and C32 was completely eliminated, and analytical results demonstrated this. The biphasic response to K1 in model C21 could still be obtained. The biphasic response to K2 of models C21 and C31 could also still be obtained. This is consistent with a demonstration of robust biphasic responses in these models, which results from a direct competing effect due to the action of K2 on two legs of the network. This is demonstrated analytically in the “Supplementary material”.

#### The landscape of biphasic responses

The landscape of biphasic responses (Table 1) can be viewed along two different axes (one in relation to the enzymes which elicit such a response, and one in relation to the requirements on the network/augmentation to achieve this). On one axis we have an entire range from biphasic response to all enzymes, to biphasic response to some enzymes, to the absence of biphasic responses. On another axis, we have different types of behaviour (ultimately tied to their origin): robust biphasic behaviour (essentially all parameters), biphasic behaviour seen even when the augmented reaction is in the unsaturated limit, to biphasic responses which depend on sequestration effects in this augmented reaction.

#### Combinations of different behaviour

**Figure 6:**
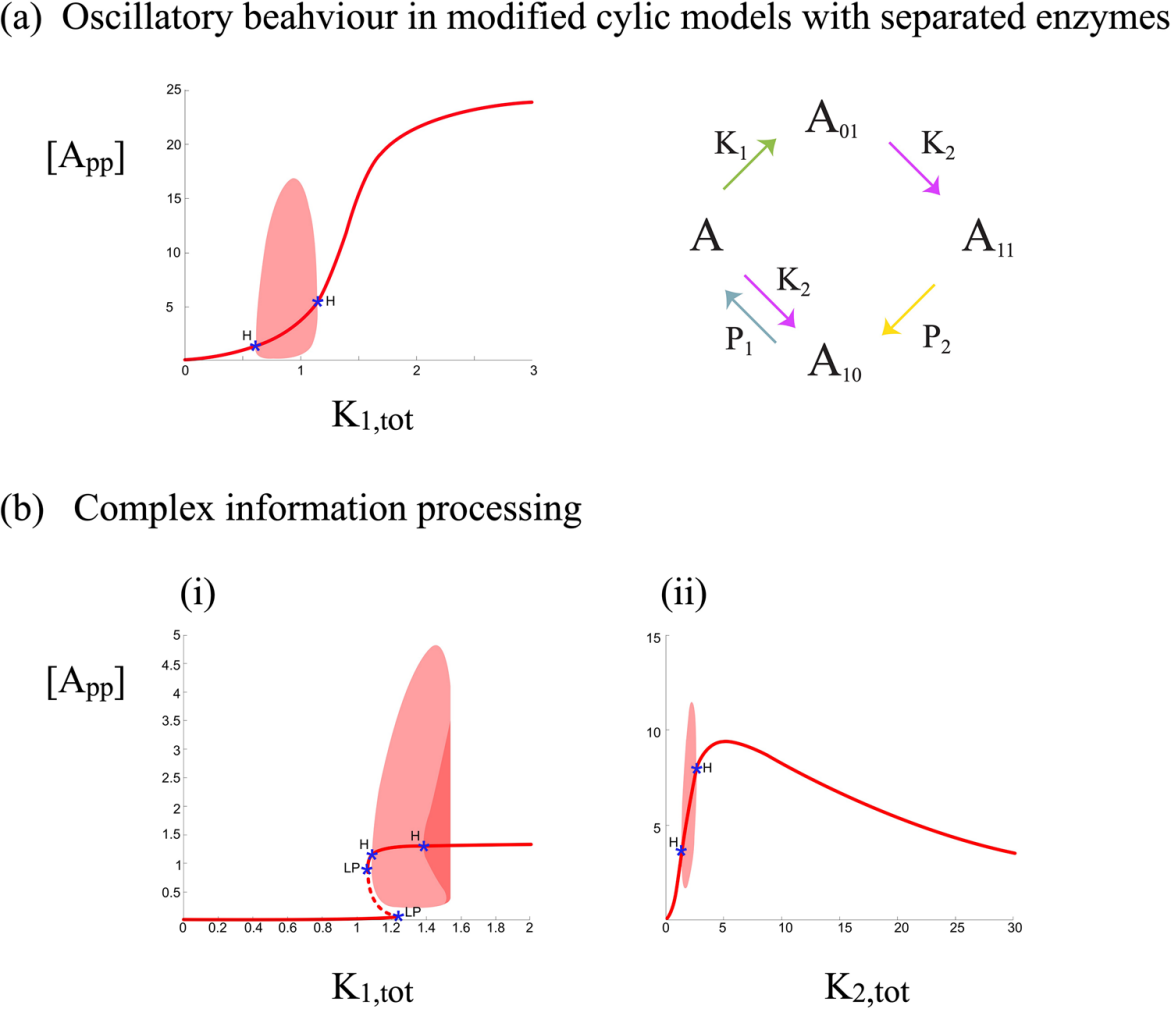
(**a**) Sustained oscillations can be seen in the cyclic phosphorylation model even with different kinase and different phosphatase with the introduction of a single kinase-mediated augmentation (model $C_{31}$). (**b**) Complex information processing can be realized in cyclic phosphorylation with a single additional enzyme-mediated step: (1) model $C_{22}$ showing a combination of bistability and oscillations and (2) model $C_{21}$ showing a combination of biphasic response and oscillations. Model $C_{21}$ also shows a combination of bistability and biphasic responses (see Fig. 5c). [Dashed and thick lines represent unstable and stable steady states respectively; LP and H represent a limit point and a Hopf bifurcation point respectively].

Our results show how a single augmentation to the cyclic mechanism can enable bistability and biphasic dose–response curves. Similarly, as seen in Fig. 6a, a single augmentation can enable oscillations in the one cyclic network which does not intrinsically exhibit it (the separate kinase, separate phosphatase case). Having isolated ingredients for each of the distinct behaviours (bistability, oscillations, biphasic responses), we find that the requirements for each of these behaviours is distinct. This prompts the question as to whether combinations of these behaviour can be seen. Figure 6b shows the case of coexistence of bistability and oscillations, as well as biphasic and oscillatory behaviour. The coexistence of bistability and biphasic behaviour is already shown in Fig. 5b. Figure S2 further demonstrates that such combinations of behaviour can even arise when the augmented reaction acts far from saturation: this is exemplified by model C31 which can exhibit a combination of bistability and oscillations, even when the augmented reaction acts far from saturation.

### Cyclic mechanisms with number of modification sites greater than two

We discuss cyclic mechanisms involving more than two modification sites. Recall that we described the cyclic mechanism in double site phosphorylation as an ordered mechanism where the order of phosphorylation and dephosphorylation are the same: this implies that the first site to be phosphorylated is the first to be dephosphorylated and the last site to be phosphorylated is the last to be dephosphorylated. This gave rise to a characteristic cyclic (irreversible) network structure seen previously, which was the basis of the observed behaviour. Now when we examine a larger number of modification sites, having the same order of phosphorylation and dephosphorylation still gives cyclic mechanisms, similar to what we have seen. However it is possible to have mechanisms where the order of phosphorylation and dephosporylation are not the same, still giving rise to cyclic networks of the type we have encountered.

#### Three modification sites

We can summarize the overall result as follows: in a three site modification system, cyclic mechanisms require either (1) the first site phosphorylated to be the first site dephosphorylated or (2) the last site phosphorylated to be the last site dephosphorylated. In only such cases do we have the phosphoforms in phosphorylation and dephosphorylations to be distinct, with the reaction network having a cyclic structure as seen above.

This can be justified as follows. Without loss of generality, assuming the order of phosphorylation involves the first site (giving rise to $A_{001}$), then the second site (giving rise to $A_{011}$) and finally the third site (giving rise to the fully modified phosphoform $A_{111}$). Now suppose the first site is dephosphorylated first, meaning that the first phosphoform in the dephosphorylation cycle is $A_{110}$. Notice that straightaway this implies, that whatever the remaining order of the dephosphorylation, the phosphoforms generated are distinct from those in the phosphorylation cycle, since they all have a zero in the last index (something none of the phosphoforms in the phosphorylation cycle have). There are two orderings for the dephosphorylation (a) $A_{111} \rightarrow A_{110} \rightarrow A_{100} \rightarrow A_{000}$ and (b) $A_{111} \rightarrow A_{110} \rightarrow A_{010} \rightarrow A_{000}$. The former represents a cyclic mechanism which is an exact analogue of the two-site mechanism studied in the paper, while the latter represents a different cyclic mechanism in its own right.

If the second site is dephosphorylated first, then there is only one possibility of a cyclic mechanism, involving the first site dephosphorylated next: this gives rise to $A_{111} \rightarrow A_{101} \rightarrow A_{100} \rightarrow A_{000}$. We notice in this case that the last site to be phosphorylated is also the last site to be dephosphorylated. Note that if the third site is dephosphorylated after the second site, this would leave a phosphoform $A_{001}$ common with the phosphorylation cycle, precluding a cyclic mechanism.

**Figure 7:**
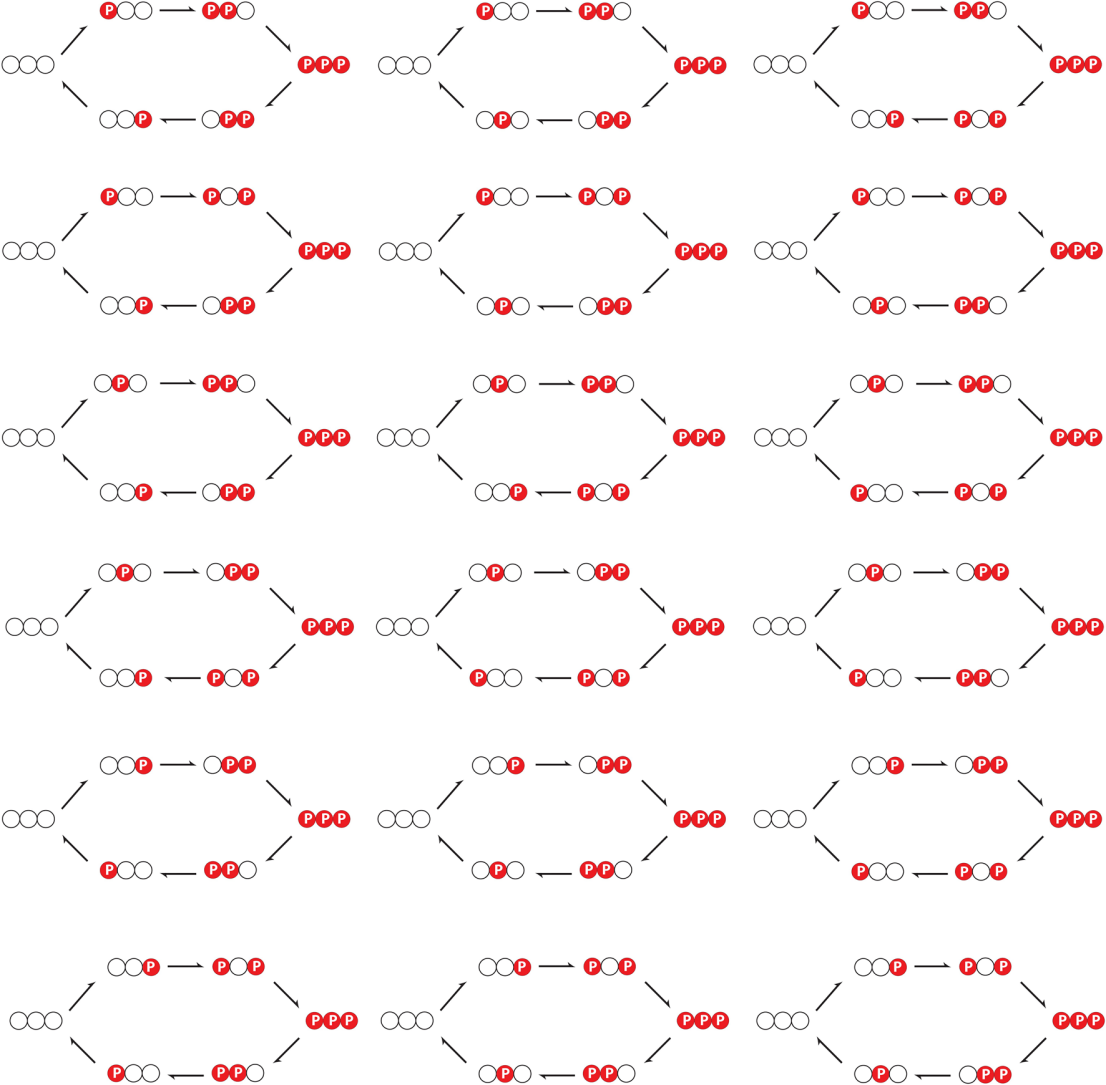
A depiction of different cyclic triple-site phosphorylation networks (see text).

Finally the third site being the first to be dephosphorylated in the dephosphorylation cycle, means that the third site phosphorylation (in the forward cycle) and dephosphorylation (in the reverse cycle) are associated with a common phosphoform. Thus no cyclic mechanism arises in this case. Figure 7 depicts a variety of three site cyclic mechanisms showing how all of them conform to the basic criterion described above.

#### A higher number of modification sites

The above characterization of the cyclic mechanism for three site modifications does not account for all cyclic mechanisms, when the number of modification sites is greater than 3. To see this, we focus on a four site modification mechanism and assume without loss of generality that the phosphorylation order is first site followed by second site followed by third site followed by fourth site. Thus the reaction pathway can be described as $A_{0000} \rightarrow A_{0001} \rightarrow A_{0011} \rightarrow A_{0111} \rightarrow A_{1111}$. Consider a dephosphorylation order which is third site followed by first site followed by fourth site followed by second site. This gives the dephosphorylation pathway as $A_{1111} \rightarrow A_{1011} \rightarrow A_{1010} \rightarrow A_{0010} \rightarrow A_{0000}$. This is an example of a cyclic mechanism, and it is clear that the partial phosphoforms are non-overlapping. Furthermore this is an example where the first site phosphorylated is not the first site dephosphorylated, and the last site phosphorylated is not the last site dephosphorylated. In fact viewed as a group, the first two or three sites phosphorylated do not correspond to the first two or three sites dephosphorylated, and likewise the last two or three sites phosphorylated is not the same as last two or three sites dephosphorylated. All in all, increasing the number of phosphorylation sites allows for multiple ways of creating cyclic mechanisms.

## Summary of analysis

Our computational results above are complemented by analytical work. Typically we use analytical approaches for questions which cannot be addressed conclusively by computations. Thus we focus on cases where particular types of behaviour are ruled out irrespective of parameters. We also use analytical work in some cases to demonstrate the presence of specific kinds of behaviour (biphasic responses, bistability).

The approach to the analysis is as follows. After writing down the steady state equations for all species (enzymes, substrates and complexes), and incorporating the conservation conditions for enzymes and substrate, a number of variables can be eliminated. This is done as follows. (1) Firstly, at steady state the concentration of complexes is proportional to the product of concentrations of the relevant (free) enzymes and substrates. (2) Using the conservation conditions for enzymes, and the expression for the complexes as discussed above, the free enzyme concentrations can be written in terms of substrate concentrations. This can be done for all relevant enzymes. (3) Substrate variables can all be written in terms of one substrate variable (say $A_{11}$). This is done by matching the net catalytic conversion to a given substrate and the net catalytic conversion away from it. This amounts to the requirement of steady state for a substrate and its enzyme substrate complex. For instance in the basic cyclic mechanism (common kinase, common phosphatase) the steady state of $A_{01}+A_{01}K$ implies that $k_{c1}[A_{00}K] =k_{c2}[A_{01}K]$. Noting point (i) above, we see immediately that at steady state $[A_{00}] \propto [A_{11}]$. Similarly by examining the steady state for $A_{11}+A_{11}P$ implies $k_{c2}[A_{01}K] =k_{c3}[A_{11}P]$. In this manner all the substrate variables can be eliminated in terms of $A_{11}$. (4) The conservation condition can now be written in terms of one substrate variable $A_{11}$. We can make a number of inferences from this.

The same approach can be used for the case of distinct kinases and/or phosphatases and also for the case of augmentations of the cyclic mechanism. In the latter case, algebraically eliminating all substrate variables may be more tedious, but through the reduction process of eliminating variables, one can obtain reduced equations to infer the relevant conclusions.

From our analytical studies we demonstrate. (1) Absence of multistationarity in cyclic mechanisms with distinct/common kinases and distinct/common phosphatases. (2) Absence of the biphasic dose response curve for $A_{11}$ in terms of specified total kinase concentrations in the basic cyclic models. (3) Absence of multistationarity for specific augmentations of the cyclic mechanism, while demonstrating its possibility in others. (4) Absence of biphasic dose response curves for specific models (C11) of the cyclic mechanism with common kinase and phosphatase, with one augmentation. (5) Absence of biphasic dose response curve for models (C22, C31, C32) of the cyclic mechanism with separate kinase and phosphatase, as the total amount of enzyme *K*1 is varied. (6) Absence of biphasic responses in models C12, C22 and C32 with respect to K or K2 when the additional reaction acted in the unsaturated limit. These various cases are studied in the “Supplementary material”.

## Conclusions

Ordering of modifications is a fundamental aspect of multisite modification and in present in a range of contexts and guises. An example involving common kinases and phosphatases in double site modification is ERK phosphorylation/dephosphorylation by MEK/MKP3 where the order of phosphorylation is the same as that of dephosphorylation^41^, giving rise to a cyclic network core. It is also known that different kinases modifying the same substrate can also involve specific ordering^50^. This is by no means restricted to the same kinase performing multiple modifications: in fact ordering can also be observed with multiple kinases^51^. Finally, the same insights also apply to dephosphorylation, which can also be ordered or random^52^.

This paper explores the impact of ordering of modifications in multisite phosphorylation and focusses on cyclic distributive double site modification models, which represent a particular ordering of modifications where the order of phosphorylation and dephosphorylation are the same, resulting in two partial phosphoforms. Cyclic mechanisms are of interest because (1) they represent a particular ordering and consequently a model system for enzymatic multisite modification, and a basic post-translation modification network and can be a reference for engineering synthetic multisite modification (2) they are found in natural systems (3) they are, from a network perspective, a bridge between ordered sequential and random mechanisms (4) analysis of this system provides and consolidates insights into the origins of different types of information processing characteristics observed in ordered and random distributive mechanisms.

**Figure 8:**
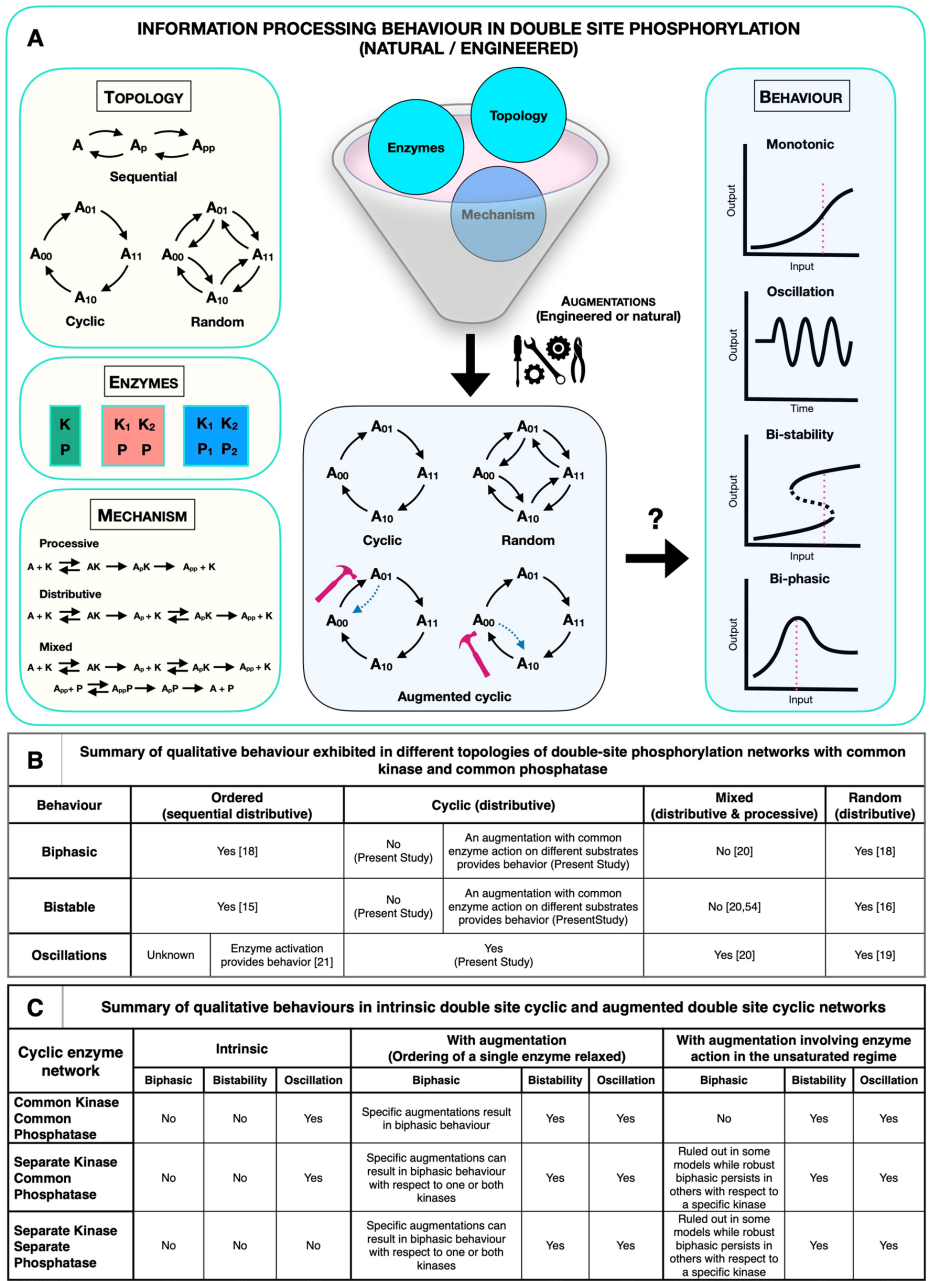
(**A**) Schematic indicates the different classes of ingredients present in multisite phosphorylation (network topology, commonality of enzymes, enzymatic mechanism). A basic question is how these ingredients determine the information processing characteristics depicted in the figure. (**B**) Summary of qualitative behavior exhibited in different classes of double site phosphorylation networks with common kinase and common phosphatase, contrasting the cyclic networks’ behavior (this paper) with those of other networks studied in literature. (**C**) Summary of qualitative behaviours shown by basic and augmented double site cyclic networks. This is established through analytical and computational work in this paper (See text and Table 1 for details).

Our goal was to characterize the presence or absence of basic information processing characteristics, specifically multistability, oscillation and biphasic responses of the maximally modified form, and thus evaluate the impact of the ordering of modifications. To do this we aim to draw a sharp boundary between the presence or absence of a given behaviour and how it depends on network interactions (and any augmentations), as well as other ingredients (commonality of enzymes, enzyme sequestration) and thus infer the minimum requirements for a given behaviour. Our analysis is performed in double site modification networks (involving different variants of common/separate kinases/phosphatases) both because of the relevance to specific contexts, and because they represent tractable networks which can be transparently analyzed to reveal basic insights which generalize to a greater number of modification sites. The results are summarized in Fig. 8.

### Oscillations

Cyclic mechanisms with a common kinase and common phosphatase or separate kinases and a common phosphatase are able to readily give oscillations. In fact, even with separate kinases and phosphatases, an extra reversible reaction in the unsaturated regime can give rise to oscillations. The former cases are strictly monostable, reinforcing an aspect seen elsewhere^20^ that oscillations can occur in networks where multistationarity is precluded for basic structural reasons.

### Bistability

Having a single reversible reaction augmenting the cyclic mechanism is sufficient for bistability. Furthermore this does not even require sufficient sequestration of enzymes/substrates in this extra reaction. All in all, a cyclic mechanism where one enzyme acts on two modification reactions, even if one of them is in the unsaturated limit, is sufficient for obtaining bistability.

### Biphasic responses

Biphasic responses in the maximally modified phosphoform stem from an in-built trade-off. This is absent in the basic cyclic mechanisms and consequently such biphasic responses are ruled out. With the presence of a single reversible reaction, we identify multiple categories of responses: (1) those where biphasic responses are ruled out for the kinase enzyme(s): in the case where there are two kinase enzymes, they are ruled out for both. In the case of two kinase enzymes, (2) those where biphasic responses are seen for one of the kinase enzymes but not the other (3) those where they are seen for both kinase enzymes. Viewed from another axes (the requirements on the augmented reaction), these biphasic responses can be categorized as either requiring the augmented reaction to be acting far from then unsaturated limit, to existing even if the augmented reaction is in the unsaturated limit, and in some of the latter cases, exhibiting robust biphasic responses. The existence of robust biphasic responses stems from a basic structural feature in the network realizing a robust trade-off (for instance the enzyme catalyzing two competing pathways).

Biphasic responses have been experimentally observed in multiple contexts^50,53–55^. Bistability and oscillations have not yet been demonstrated for the basic modification systems in isolation, but are predicted from very basic considerations.

### Contrast with other mechanisms

Ordered sequential models with purely processive mechanisms of enzymatic modification result in a single globally stable steady state. The ordered sequential model with distributive modification can result in bistability with one or both enzymes being common to the different modifications. Ordered sequential models with common kinases and phosphatases can generate biphasic responses. Oscillations have thus far been elusive. The random mechanism readily provides multistability and oscillations, with separate/common kinases and separate/common phosphatases^21^.

### Cyclic mechanisms and ordered mixed mechanisms

The ordered sequential mechanism with mixed mechanisms of modification (one direction of modification is distributive and the other processive), share a common characteristic of the basic cyclic mechanisms, of having a single steady state which can lose stability to a Hopf bifurcation. There too, biphasic responses are ruled out. The effective separation of phosphorylation and dephosphorylation legs (owing to the partial phosphoform released in only one leg in the mixed mechanism), is a common feature of the two systems. We find oscillations possible in the cyclic system even when the binding constants of the partial phosphoforms to relevant enzymes, is relatively low. If these binding constants for one of the partial phosphoforms were high, then the relevant partial phosphoform would hardly be present in free form (assuming moderate unbinding rates and sufficient enzyme), a feature reminiscent of the processive leg of the mixed mechanism model. We thus conclude that oscillations in the cyclic mechanism are not merely a simple echo of those in mixed mechanisms.

### Cyclic mechanisms vs ordered sequential and random mechanisms

The contrast with the ordered sequential distributive model shows straightaway that the presence of different phosphoforms, eliminates a trade-off responsible for biphasic responses, and also multistationarity, but interestingly that this very feature can readily generate oscillations. The contrast with random modification mechanisms highlights that behaviour such as bistability and oscillations actually requires only a small number of underlying factors present in the random mechanism to achieve. It also straightaway points to multiple regions/corners in the parameter space where such behaviour is achieved.

### The cyclic mechanism as an enabler of bistability and oscillations with minimal ingredients

Non-trivial dynamical characteristics such as bistability and oscillations in multisite modification ultimately rely on (1) a common enzyme in more than one modification step and (2) sequestration of enzymes in complexes, which is the source of non-linearity. Our analysis of cyclic mechanisms with a single augmented step in an unsaturated regime shows that it is possible (separate kinase, separate phosphatase case) to realize both bistability and oscillations in networks with a single common enzyme acting in only two steps, with one of them in the unsaturated limit. This represents a minimum combination of the factors outlined above. Random mechanisms have more than one enzyme acting in multiple steps, while sequential ordered mechanisms (1) need at least one common enzyme for bistability and (2) have not been shown to exhibit oscillations intrinsically even with greater degrees of coupling of enzymes and sequestration.

The above results are relevant to both systems and synthetic biology, and their intersection. All the types of basic behaviour considered here (oscillations, multistability, biphasic responses) are being investigated experimentally in either natural or engineered contexts involving multisite modification.

### Systems biology

Since multisite modification systems are part of signalling networks, there is a need to understand in detail (1) the intrinsic characteristics of multisite modification and (2) how they function as part of signalling pathways. In the context of (1), analyzing cyclic mechanisms is of interest, because it is encountered naturally (as seen, for example in^37^, where the compromising of phosphorylation is associated with certain disorders), and is also an example of different basic circuit containing multisite phosphorylation. It provides insights into the behaviour of circuits (for example random modification mechanisms), which we discuss below. In addition, it also provides insights into variants or augmentations of multisite modification (e.g. phosphorylation dephosphorylation in different compartments, with intermediate phosphoform not shuttling, or one of the modification directions involving a scaffold), which exhibit a similar chemical modification logic.

### A bottom-up approach to understanding behaviour of random mechanisms

Random mechanisms of multisite modification with different degrees of commonality of enzymes are fundamental PTM circuits. Characterizing their behaviour, their dependence on parameters and “design principles” underlying different characteristic behaviour they exhibit is fundamental to understanding multisite modification systems in systems biology. While some aspects can be studied to an extent by exhaustive parametric scanning, understanding the sources and origins of different behaviour needs a much more concerted effort and prompts multiple questions. Do these arise from dominant interactions/subnetworks? Do they arise from non-trivial interactions between different sub-networks? What type of sequestration effects are important? What impact do these aspects have on the robustness of behaviour? As part of this effort, one needs to consider stripped down versions of the full random mechanism, where it is possible to characterize both the presence and absence of behaviour, as well as parametric dependence clearly. Cyclic mechanisms (with minimal augmentations) represent extreme stripped down versions in this regard. They represent tractable starting points from which augmentations can be added step by step (towards full random mechanisms), along with a clearer characterization of parametric dependence in these simpler networks at every stage. Such a bottom-up approach implemented in stages can provide a distinct systematic way of understanding in depth the behaviour and its origins in random mechanisms. In this context, we also point out that the ERK phosphorylation/dephosphorylation by MEK/MKP3, involves a specific ordering giving rise to a cyclic network core, in addition to which other reactions are considered^15,30,42^. An approach of the type outlined allows for the systematic elucidation of additional layers overlaid on the cyclic network core (which in turn is determined by the ordering of the modifications), with respect to the introduction of new information processing characteristics.

### Synthetic biology

Viewed from a bottom-up synthetic chemical approach, we are able to isolate fairly simple circuits which can in principle, be engineered to achieve circuit behaviour. From the perspective of building life to understand it^56^, we can say that the construction of such circuits in-silico is an example of building basic ingredients with a view towards understanding the origins of different kinds of behaviour in more complex post-translational modification circuits. The engineering of multisite phosphorylation in synthetic biology is being pursued in multiple directions experimentally^43,44^, and this in turn is one aspect of the engineering of reaction networks^57,58^. Our analysis shows that separating phosphorylation/dephosphorylation steps is a key enabler of oscillations. Implementing such a design principle (for example through the synthetic deployment of scaffolds for phosphorylation/dephosphorylation, or ensuring these happen in different compartments), could facilitate the creation of oscillatory circuits in cellular and cell-free systems. Our analysis in this paper also provides a template for the engineering of different circuits with different combinations of information processing characteristics.

### Chemical information processing

The results are also relevant to chemical information processing more generally. While complex behaviour in chemical pathways and circuits has been long established, the fact that such behaviour can occur in more basic chemical modification scenarios, with the crucial role of species sequestration, is of more recent vintage. In such systems, determining the effect of sequestration and how it combines with basic chemical modification mechanisms, as well as the underlying modification network has led to multiple basic unanswered questions. By examining very basic modification networks and circuits therein, we are able to more sharply define the interplay between network ingredients, topology and sequestration to achieve certain behaviour. In this regard, being able to rule out the presence of such behaviour in circuits independent of parameters is important, as it allows us to more sharply draw the boundary between the presence and absence of such behaviour. Further analysis can build on this to examine robustness of behaviour and how additional ingredients contribute to this. The role of different ingredients such as the commonality or distinctness of modification enzymes and how they contribute to the tuneability of the circuits is another important aspect. The realization of new chemical circuits to achieve different information processing tasks (including chemical computing) is being pursued in a range of non-biological avenues as well^59–61^.

All in all, the results are relevant to understanding important aspects of the chemical underpinning of signalling circuits and their complexity, how they can be used to building new information processing circuits, and also in emerging areas which aim to bridge the living and the non-living, with a chemical focus.

## Supplementary information

Supplementary Information 1

Supplementary Information 2

Supplementary Information 3

Supplementary Information 4

Supplementary Information 5
