# Supplementary figures and images for: Exploring cyclic networks of multisite modification reveals origins of information processing characteristics
Source: Sci Rep. 2020 Oct 6;10:16542. doi: 10.1038/s41598-020-73045-9 (PMC7539153; doi:10.1038/s41598-020-73045-9)

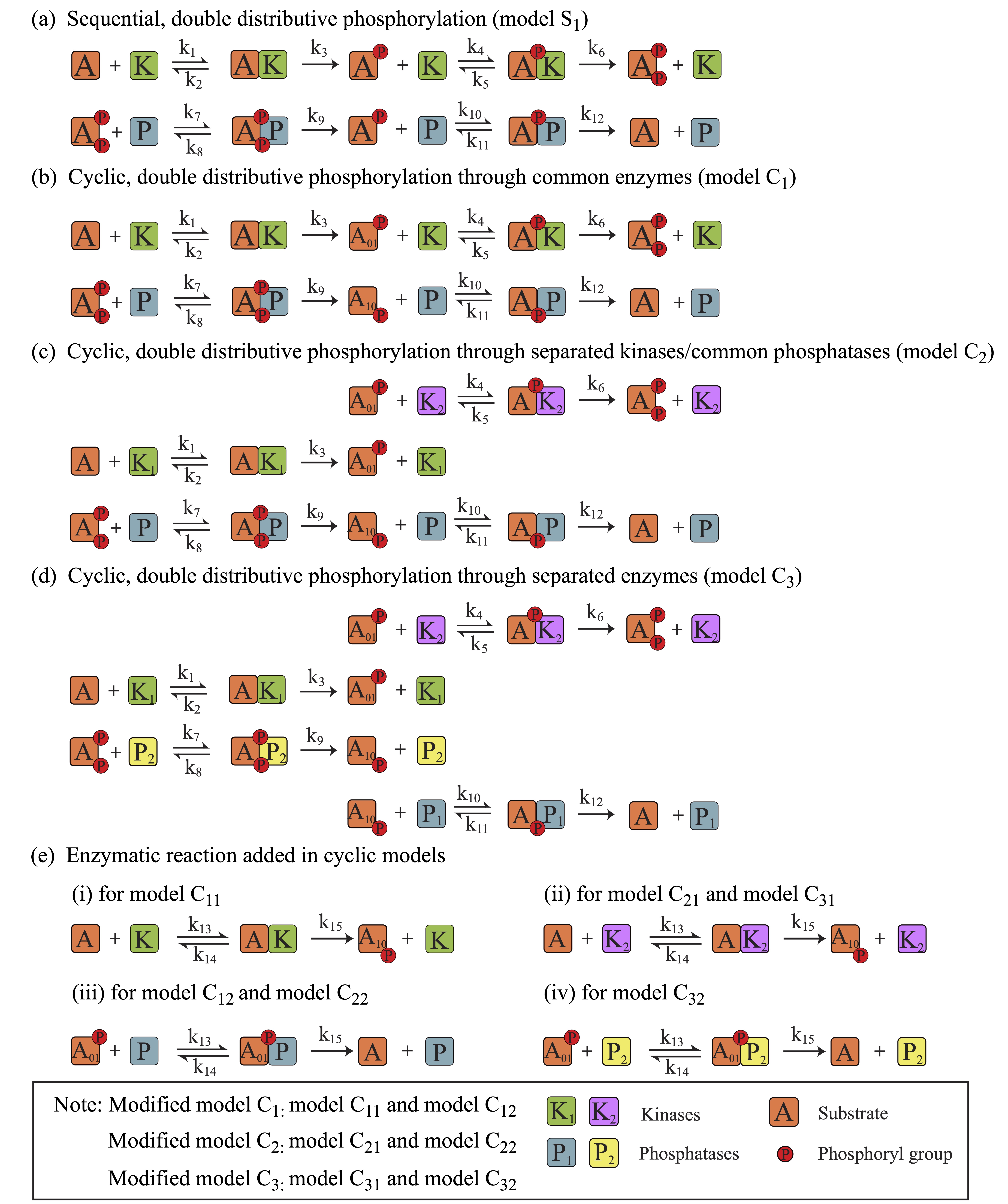

Supplement: Supplementary file 1 — Supplementary Information 1 [file 41598_2020_73045_MOESM1_ESM.jpg]

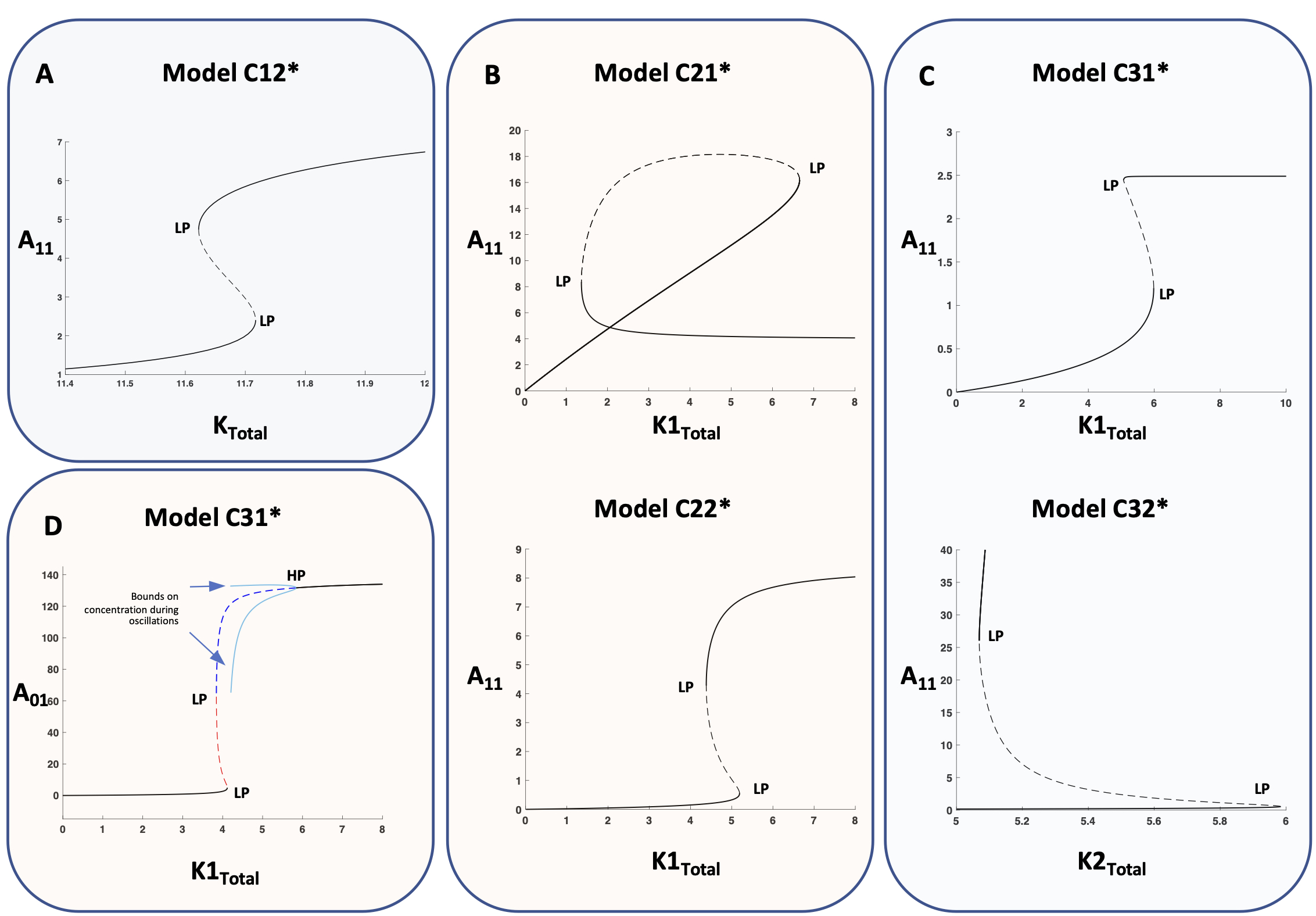

Supplement: Supplementary file 2 — Supplementary Information 2 [file 41598_2020_73045_MOESM2_ESM.jpg]

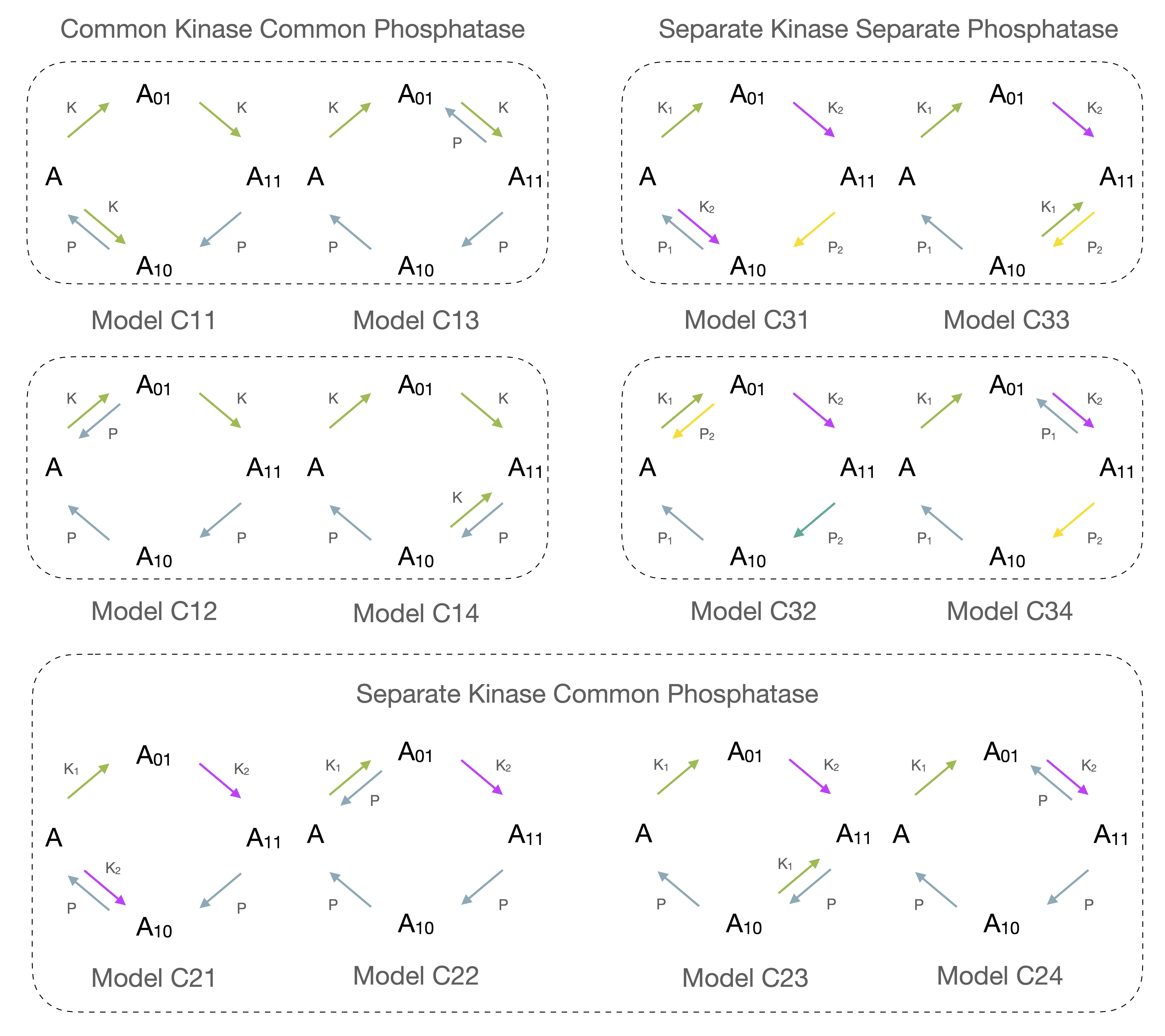

Supplement: Supplementary file 3 — Supplementary Information 3 [file 41598_2020_73045_MOESM3_ESM.jpg]

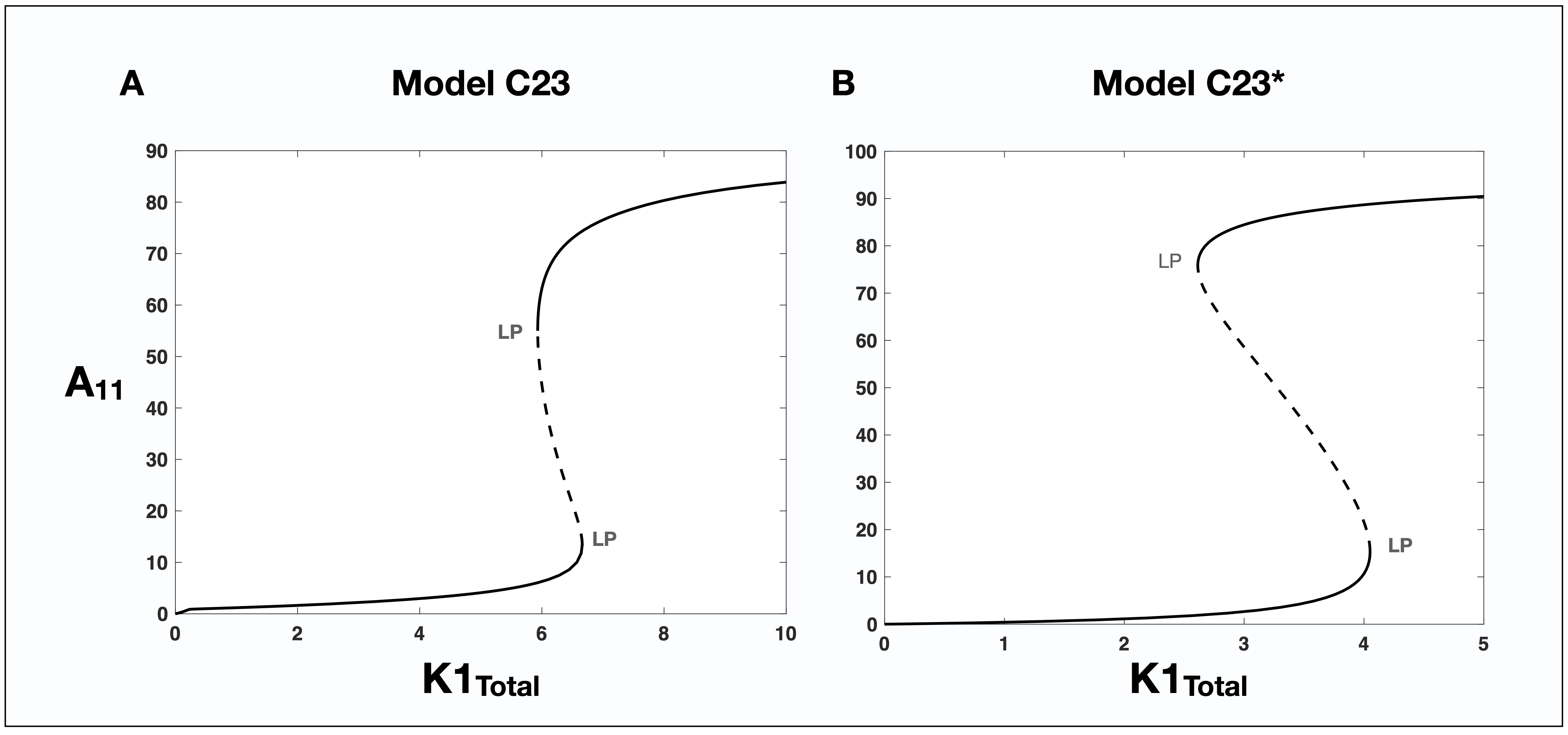

Supplement: Supplementary file 4 — Supplementary Information 4 [file 41598_2020_73045_MOESM4_ESM.jpg]
