# Supplementary material for: Exploring cyclic networks of multisite modification reveals origins of information processing characteristics
Source: Sci Rep. 2020 Oct 6;10:16542. doi: 10.1038/s41598-020-73045-9 (PMC7539153; doi:10.1038/s41598-020-73045-9)
Supplement: Supplementary file 5 — Supplementary Information 5 [file 41598_2020_73045_MOESM5_ESM.pdf]

Exploring cyclic networks of multisite modification reveals origins of information processing characteristics

Thapanar Suwanmajo [1,2], Vaidhiswaran Ramesh [3] and J. Krishnan [3,4]

[1] Center of Excellence in Materials Science and Technology, Chiang Mai University, Chiang Mai 50200, Thailand.
[2] Department of Chemistry, Faculty of Science, Chiang Mai University, Chiang Mai 50200, Thailand.
[3] Department of Chemical Engineering, Centre for Process Systems Engineering, Imperial College London, London, SW7 2AZ, UK.
[4] Institute for Systems and Synthetic Biology, Imperial College London, South Kensington Campus, London SW7 2AZ, UK.
j.krishnan@imperial.ac.uk. Ph: 44-20-7594-6633; Fax: 44-20-7594-6606.

In this Supplementary material, we present a number of details related to the material presented in the main text. We present the ODE model equations for the main models, and discuss in turn analytical results pertaining to both multistationarity/bistability and biphasic responses in various models. Supporting computational results are also presented. We also provide information into parameters used in the main text.

1 Models

We present the kinetic models employed in our study. In general the kinetic models are developed by describing enzymatic modification of the substrate in standard way: binding reversibly to the substrate to form a complex which irreversibly dissociates to give the product and release the enzyme. Whenever an enzyme effects multiple modifications, it is assumed to act distributively. The basic cyclic models (C1, C2, C3) correspond to cyclic networks described in the main text (Fig. 1). The equations for a 2-site cyclic distributive mechanism, model C1 (Fig. 7), which corresponds to a cylic network with a common kinase and a common phosphatase effecting the modifications, are:

$$
\begin{aligned}
d[A]/dt &= k_2[AK] - k_1[A][K] + k_{12}[A_{10}P] \\
d[K]/dt &= (k_2+k_3)[AK] - k_1[A][K] + (k_5+k_6)[A_{01}K] - k_4[A_{01}][K] \\
d[AK]/dt &= k_1[A][K] - (k_2+k_3)[AK] \\
d[A_{01}]/dt &= k_3[AK] - k_4[A_{01}][K] + k_5[A_{01}K] \\
d[A_{01}K]/dt &= k_4[A_{01}][K] - (k_5+k_6)[A_{01}K] \\
d[A_{11}]/dt &= k_6[A_{01}K] + k_8[A_{11}P] - k_7[A_{11}][P] \\
d[P]/dt &= (k_8+k_9)[A_{11}P] - k_7[A_{11}][P] + (k_{11}+k_{12})[A_{10}P] - k_{10}[A_{10}][P] \\
d[A_{10}]/dt &= k_9[A_{11}P] - k_{10}[A_{10}][P] + k_{11}[A_{10}P] \\
d[A_{11}P]/dt &= k_7[A_{11}][P] - (k_8+k_9)[A_{11}P] \\
d[A_{10}P]/dt &= k_{10}[A_{10}][P] - (k_{11}+k_{12})[A_{10}P] \qquad (1)
\end{aligned}
$$

This is simply the mathematical description of the network in Fig. 1(a). In an analogous way, the equations for model C2 (see Fig. 1 for a schematic) which corresponds to a cyclic 2 site modification network with different kinases and a common phosphatase effecting the (de) modifications are:

$$
\begin{aligned}
d[A]/dt &= k_2[AK_1] - k_1[A][K_1] + k_{12}[A_{10}P] \\
d[K_1]/dt &= (k_2+k_3)[AK_1] - k_1[A][K_1] \\
d[K_2]/dt &= (k_5+k_6)[A_{01}K_2] - k_4[A_{01}][K_2] \\
d[AK_1]/dt &= k_1[A][K_1] - (k_2+k_3)[AK_1] \\
d[A_{01}]/dt &= k_3[AK_1] - k_4[A_{01}][K_2] + k_5[A_{01}K_2] \\
d[A_{01}K_2]/dt &= k_4[A_{01}][K_2] - (k_5+k_6)[A_{01}K_2] \\
d[A_{11}]/dt &= k_6[A_{01}K_2] + k_8[A_{11}P] - k_7[A_{11}][P] \\
d[P]/dt &= (k_8+k_9)[A_{11}P] - k_7[A_{11}][P] + (k_{11}+k_{12})[A_{10}P] - k_{10}[A_{10}][P] \\
d[A_{10}]/dt &= k_9[A_{11}P] - k_{10}[A_{10}][P] + k_{11}[A_{10}P] \\
d[A_{11}P]/dt &= k_7[A_{11}][P] - (k_8+k_9)[A_{11}P] \\
d[A_{10}P]/dt &= k_{10}[A_{10}][P] - (k_{11}+k_{12})[A_{10}P] \qquad (2)
\end{aligned}
$$

The only difference (from model C1) is that there are different kinases involved in the phosphorylation leg of the cycle: K_1 phosphorylates A while K_2 phosphorylates A_{01}. The common phosphatase dephosphorylates A_{11} and A_{10}

Model C3 differs from C2 in that there are different phosphatases involved in the dephosphorylation leg. Phosphatase P_2 dephosphorylates A_{11} while phosphatase P_1 dephosphorylates A_{10}. Other than this, the model is identical to C2. The equations for model C3 are (see Fig. 1 for a schematic):

$$
\begin{aligned}
d[A]/dt &= k_2[AK_1] - k_1[A][K_1] + k_{12}[A_{10}P_1] \\
d[K_1]/dt &= (k_2 + k_3)[AK_1] - k_1[A][K_1] \\
d[K_2]/dt &= (k_5 + k_6)[A_{01}K_2] - k_4[A_{01}][K_2] \\
d[AK_1]/dt &= k_1[A][K_1] - (k_2 + k_3)[AK_1] \\
d[A_{01}]/dt &= k_3[AK_1] - k_4[A_{01}][K_2] + k_5[A_{01}K_2] \\
d[A_{01}K_2]/dt &= k_4[A_{01}][K_2] - (k_5 + k_6)[A_{01}K_2] \\
d[A_{11}]/dt &= k_6[A_{01}K_2] + k_8[A_{11}P_2] - k_7[A_{11}][P_2] \\
d[P_2]/dt &= (k_8 + k_9)[A_{11}P_2] - k_7[A_{11}][P_2] \\
d[P_1]/dt &= (k_{11} + k_{12})[A_{10}P_1] - k_{10}[A_{10}][P_1] \\
d[A_{10}]/dt &= k_9[A_{11}P_2] - k_{10}[A_{10}][P_1] + k_{11}[A_{10}P_1] \\
d[A_{11}P_2]/dt &= k_7[A_{11}][P_2] - (k_8 + k_9)[A_{11}P_2] \\
d[A_{10}P_1]/dt &= k_{10}[A_{10}][P_1] - (k_{11} + k_{12})[A_{10}P_1] \qquad (3)
\end{aligned}
$$

Models of cyclic networks with additional reactions. We now present the equations for models which build on the basic cyclic models above with an additional reaction, either kinase-mediated or phosphatase-mediated (see Fig. 1(c)).

Model C21, builds on the basic model C2 (different kinase common phosphatase) with an additional reaction mediated by kinase K_2, involving the conversion of A to A_{10} (note that the phosphorylation activity of $K_2$is associated with the first index of the subscript). The equations for model C21 are

$$
\begin{aligned}
d[A]/dt &= k_2[AK_1] - k_1[A][K_1] + k_{12}[A_{10}P] + k_{14}[AK_2] - k_{13}[A][K_2] \\
d[K_1]/dt &= (k_2 + k_3)[AK_1] - k_1[A][K_1] \\
d[K_2]/dt &= (k_5 + k_6)[A_{01}K_2] - k_4[A_{01}][K_2] - k_{13}[A][K_2] + (k_{14} + k_{15})[AK_2] \\
d[AK_1]/dt &= k_1[A][K_1] - (k_2 + k_3)[AK_1] \\
d[A_{01}]/dt &= k_3[AK_1] - k_4[A_{01}][K_2] + k_5[A_{01}K_2] \\
d[A_{01}K_2]/dt &= k_4[A_{01}][K_2] - (k_5 + k_6)[A_{01}K_2] \\
d[A_{pp}]/dt &= k_6[A_{01}K_2] + k_8[A_{pp}P] - k_7[A_{pp}][P] \\
d[P]/dt &= (k_8 + k_9)[A_{pp}P] - k_7[A_{pp}][P] + (k_{11} + k_{12})[A_{10}P] - k_{10}[A_{10}][P] \\
d[A_{10}]/dt &= k_9[AppP] - k_{10}[A_{10}][P] + k_{11}[A_{10}P] + k_{15}[AK_2] \\
d[A_{pp}P]/dt &= k_7[A_{pp}][P] - (k_8 + k_9)[A_{pp}P] \\
d[A_{10}P]/dt &= k_{10}[A_{10}][P] - (k_{11} + k_{12})[A_{10}P] \\
d[AK_2]/dt &= k_{13}[A][K_2] - (k_{14} + k_{15})[AK_2] \qquad (4)
\end{aligned}
$$

As can be seen here, there are only a small number of changes compared to the model C2: the presence of a new complex AK_2 (and its associated equations), the presence of the effect of A reversibly binding to K_2 (seen in the equations for $[A]$ and $[K_2]$, and the effect of AK_2 in the equation for $[A_{10}]$.

Model C22 incorporates an additional dephosphorylation of A_{01} to A, mediated by P (the common phosphatase). This results in an additional complex $A_{01}P$ and its associated equations, additional relevant terms in the equations for $[A_{01}]$,$[P]$, along with the effect of $A_{01}P$ on A. The equations for model C22 are

$$
\begin{aligned}
d[A]/dt &= k_2[AK_1] - k_1[A][K_1] + k_{12}[A_{10}P] + k_{15}[A_{01}P] \\
d[K_1]/dt &= (k_2 + k_3)[AK_1] - k_1[A][K_1] \\
d[K_2]/dt &= (k_5 + k_6)[A_{01}K_2] - k_4[A_{01}][K_2] \\
d[AK_1]/dt &= k_1[A][K_1] - (k_2 + k_3)[AK_1] \\
d[A_{01}]/dt &= k_3[AK_1] - k_4[A_{01}][K_2] + k_5[A_{01}K_2] + k_{14}[A_{01}P] - k_{13}[A_{01}][P] \\
d[A_{01}K_2]/dt &= k_4[A_{01}][K_2] - (k_5 + k_6)[A_{01}K_2] \\
d[A_{pp}]/dt &= k_6[A_{01}K_2] + k_8[A_{pp}P] - k_7[A_{pp}][P] \\
d[P]/dt &= (k_8 + k_9)[A_{pp}P] - k_7[A_{pp}][P] + (k_{11} + k_{12})[A_{10}P] - k_{10}[A_{10}][P] - k_{13}[A_{01}][P] + (k_{14} + k_{15})[A_{01}\text{[illegible]} \\
d[A_{10}]/dt &= k_9[AppP] - k_{10}[A_{10}][P] + k_{11}[A_{10}P] \\
d[A_{pp}P]/dt &= k_7[A_{pp}][P] - (k_8 + k_9)[A_{pp}P] \\
d[A_{10}P]/dt &= k_{10}[A_{10}][P] - (k_{11} + k_{12})[A_{10}P] \\
d[A_{01}P]/dt &= k_{13}[A_{01}][P] - (k_{14} + k_{15})[A_{01}P] \qquad (5\text{[illegible]}
\end{aligned}
$$

In an exactly analogous way the equations for models C31 and C32 are obtained. The augmentations for C31 and C32 are the same as C21 and C22 respectively, and the only difference is that these augmentations are built into model C3 (different kinase and different phosphatase) rather than model C2.

The equations for model C31 are

$$
\begin{aligned}
d[A]/dt &= k_2[AK_1] - k_1[A][K_1] + k_{12}[A_{10}P_1] - k_{13}[A][K_2] + k_{14}[AK_2] \\
d[K_1]/dt &= (k_2 + k_3)[AK_1] - k_1[A][K_1] \\
d[K_2]/dt &= (k_5 + k_6)[A_{01}K_2] - k_4[A_{01}][K_2] - k_{13}[A][K_2] + (k_{14} + k_{15})[AK_2] \\
d[AK_1]/dt &= k_1[A][K_1] - (k_2 + k_3)[AK_1] \\
d[A_{01}]/dt &= k_3[AK_1] - k_4[A_{01}][K_2] + k_5[A_{01}K_2] \\
d[A_{01}K_2]/dt &= k_4[A_{01}][K_2] - (k_5 + k_6)[A_{01}K_2] \\
d[A_{pp}]/dt &= k_6[A_{01}K_2] + k_8[A_{pp}P_2] - k_7[A_{pp}][P_2] \\
d[P_2]/dt &= (k_8 + k_9)[A_{pp}P_2] - k_7[A_{pp}][P_2] \\
d[P_1]/dt &= (k_{11} + k_{12})[A_{10}P_1] - k_{10}[A_{10}][P_1] \\
d[A_{10}]/dt &= k_9[AppP_2] - k_{10}[A_{10}][P_1] + k_{11}[A_{10}P_1] + k_{15}[AK_2] \\
d[A_{pp}P_2]/dt &= k_7[A_{pp}][P_2] - (k_8 + k_9)[A_{pp}P_2] \\
d[A_{10}P_1]/dt &= k_{10}[A_{10}][P_1] - (k_{11} + k_{12})[A_{10}P_1] \\
d[AK_2]/dt &= k_{13}[A][K_2] - (k_{14} + k_{15})[AK_2] \qquad (6)
\end{aligned}
$$

The equations for model C32 are

$$
\begin{aligned}
d[A]/dt &= k_2[AK_1] - k_1[A][K_1] + k_{12}[A_{10}P_1] + k_{15}[A_{01}P_2] \\
d[K_1]/dt &= (k_2 + k_3)[AK_1] - k_1[A][K_1] \\
d[K_2]/dt &= (k_5 + k_6)[A_{01}K_2] - k_4[A_{01}][K_2] \\
d[AK_1]/dt &= k_1[A][K_1] - (k_2 + k_3)[AK_1] \\
d[A_{01}]/dt &= k_3[AK_1] - k_4[A_{01}][K_2] + k_5[A_{01}K_2] - k_{13}[A_{01}][P_2] + k_{14}[A_{01}P_2] \\
d[A_{01}K_2]/dt &= k_4[A_{01}][K_2] - (k_5 + k_6)[A_{01}K_2] \\
d[A_{pp}]/dt &= k_6[A_{01}K_2] + k_8[A_{pp}P_2] - k_7[A_{pp}][P_2] \\
d[P_2]/dt &= (k_8 + k_9)[A_{pp}P_2] - k_7[A_{pp}][P_2] - k_{13}[A_{01}][P_2] + (k_{14} + k_{15})[A_{01}P_2] \\
d[P_1]/dt &= (k_{11} + k_{12})[A_{10}P_1] - k_{10}[A_{10}][P_1] \\
d[A_{10}]/dt &= k_9[AppP_2] - k_{10}[A_{10}][P_1] + k_{11}[A_{10}P_1] \\
d[A_{pp}P_2]/dt &= k_7[A_{pp}][P_2] - (k_8 + k_9)[A_{pp}P_2] \\
d[A_{10}P_1]/dt &= k_{10}[A_{10}][P_1] - (k_{11} + k_{12})[A_{10}P_1] \\
d[A_{01}P_2]/dt &= k_{13}[A_{01}][P_2] - (k_{14} + k_{15})[A_{01}P_2] \qquad (7)
\end{aligned}
$$

In a similar way, the equations for models C11 and C22 are obtained (not shown). They represent the same augmentations as C21 and C22 respectively, overlaid on model C1 (see Fig. 1).

Comments on other augmentations to cyclic networks. We have listed 6 models involving either an additional kinase reaction augmentation (3 cases) or an additional phosphatase reaction augmentation (3 cases). Clearly, for a given basal cyclic network, there are 4 possibilities for a single augmentation. This gives rise to an additional 6 networks (see Fig. S3). For the most part, we do not study these additional cases in detail except to note that: (i) The networks studied in the main text considered representative cases involving an augmentation by a kinase mediated reaction or a phosphatase-mediated reaction: taken together this already reveals the essential landscape of behaviour and possibilities, from the focal point of our study. (ii) The additional models can be analyzed in analogous terms to the cases studied. (iii) From the perspective of bistability and oscillations, 4 of these additional networks (the common kinase common phosphatase and the separate kinase separate kinase cases) exactly map onto networks we have studied in the main text. (iv) Essential results and insights from the remaining two are discussed below in the context of results. (v) With regard to biphasic dose responses the models we have studied in the main text already reveal the landscape and types of responses which may be seen.

2 Bistability in cyclic mechanisms and variants

The text discusses multiple instances of the presence or absence of bistability (and multistationarity in general) in different cyclic models and their variants. Here we use analytical approaches to demonstrate that bistability can be ruled out in some of these cases, irrespective of parameters. Specifically we show that (i) All the basic cyclic models (C1,C2,C3) possess only a single biologically feasible steady state. (ii) Specific augmentations of the cyclic models, such as model C11 also possess only one steady state. This complements computational results which directly demonstrates the presence of bistability in other models.

Our analysis of the models employs a number of basic points repeatedly, and we summarize them here. We make a comment on notation in the analysis. Here and below we use $[A_{00}]$ to denote the concentration of the unphosphorylated form of substrate, which is referred to as $[A]$ above. Additionally we refer to the catalytic constants of modification of $A_{00}, A_{01}, A_{11}, A_{10}$ as $k_{c1}, k_{c2}, k_{c3}, k_{c4}$ respectively. Naturally this specific notation has no consequence for the analysis and the results which emerge therefrom.

The approach to the analysis is as follows. After writing down the steady state equations for all species (enzymes, substrates and complexes), and incorporating the conservation conditions for enzymes and substrate, a number of variables can be eliminated. This is done as follows. (i) Firstly, at steady state the concentration of complexes is proportional to the product of concentrations of the relevant (free) enzymes and substrates. This point is repeatedly invoked in the analysis below. (ii) Using the conservation conditions for enzymes, and the expression for the complexes as discussed above, the free enzyme concentrations can be written in terms of substrate concentrations. This can be done for all relevant enzymes. (iii) The concentration of different substrates can all be written in terms of the concentration of one substrate (say A_{11}). This is done by matching the net catalytic conversion to a given substrate and the net catalytic conversion away from it. This amounts to the requirement of steady state for a substrate and its enzyme substate complex. For instance in the basic cyclic model C1 (common kinase, common phosphatase) the steady state of $A_{01} + A_{01}K$ implies that $k_{c1}[A_{00}K] = k_{c2}[A_{01}K]$. Noting point (i) above, we see immediately that at steady state $[A_{00}] \propto [A_{01}]$. Similarly by examining the steady state for $A_{11} + A_{11}P$ implies $k_{c2}[A_{01}K] = k_{c3}[A_{11}P]$. A steady state for $[A_{00}] + [A_{00}K]$ yields $k_{c1}[A_{00}K] = k_{c4}[A_{10}P]$. In this manner all the substrate variables can be eliminated in terms of A_{11}. (iv) The substrate conservation condition can now be written in terms of one substrate variable A_{11}. We can make a number of inferences from this.

Cyclic Model C1. Since concentrations of complexes at steady state is proportional to the product of

the relevant free enzyme and substrate we note that at steady state: (i) By examining the steady state of $A_{01} + A_{01}K$, we have $[A_{00}K] \propto [A_{01}K]$ which implies that $[A_{00}] \propto [A_{01}]$. This uses the fact that at steady state the concentration of a complex is proportional to the product of the enzyme and substrate concentrations (point (i) in the previous paragraph: hereafter we use this repeatedly without explicitly referencing this point). By an exactly analogous analysis of the dephosphorylation cycle (examining steady state of $A_{10} + A_{10}P$, we have $[A_{11}] \propto [A_{10}]$. Notice that here and below, proportionality implies involving proportionality constants which are purely (positive) functions of kinetic constants and total enzyme/substrate concentrations. (ii) By examining the steady state of $A_{01} + A_{01}K + A_{11} + A_{11}P$ we have $[A_{00}K] \propto [A_{11}P]$. It follows that $[A_{00}] \propto [A_{11}]P/K$. (iii) From a conservation of phosphatase, we find that the free phosphatase$[P] = P_{tot}/(1 + \alpha[A_{11}] + \beta[A_{10}])$, where α, β are positive constants. From point (i), we have $P = P_{tot}/(1 + \gamma[A_{11}])$, where γ is a positive constant. In a similar way by examining the conservation of kinase we have $[K] = K_{tot}/(1 + \alpha_1[A_{00}] + \beta_1[A_{10}])$, which again from point (i) can be simplified to an expression of the form $[K] = K_{tot}/(1 + \gamma_1[A_{00}])$. (iii) Finally $[A_{00}K] \propto [A_{11}P]$ implies that $a_1[A_{00}]/(1 + \gamma_1[A_{00}]) = b_1[A_{11}]/(1 + \gamma[A_{11}])$ for suitable positive constants a_1, b_1. By taking the inverse of each side, we have $\gamma_1/a_1 + (1/a_1)(1/[A_{00}]) = \gamma/b_1 + (1/b_1)(1/[A_{11}])$. This directly implies that one of these concentrations can be expressed as a monotonically increasing function of the other. For instance if $\gamma/b_1 > \gamma_1/a_1$, $[A_{00}] \propto [A_{11}]/(c_1 + [A_{11}])$, while if $\gamma/b_1 < \gamma_1/a_1$ then $[A_{11}] \propto [A_{00}]/(c_2 + [A_{00}])$, where c_1, c_2 are positive constants. (iv) Now we use the overall substrate conservation condition $[A_{00}] + [A_{00}K] + [A_{01}] + [A_{01}K] + [A_{11}] + [A_{11}P] + [A_{10}] + [A_{10}P] = A_{tot}$. Here, note that intermediate phosphoforms are proportional to either $[A_{00}]$ or $[A_{11}]$, all kinase complexes are proportional to $[A_{00}K]$ which in turn is proportional to $[A_{00}]/(1 + \gamma[A_{00}])$, and all phosphatase complexes are proportional to $[A_{11}P]$ which in turn is proportional to $[A_{11}]/(1 + \gamma_1[A_{11}])$. Furthermore either $[A_{00}] \propto [A_{11}]/(c_1 + [A_{11}])$ or $[A_{11}] \propto [A_{00}]/(c_2 + [A_{00}])$. In the former case, all variables can be eliminated in terms of $[A_{11}]$. Note that all kinase complexes are proportional, and proportional to $[A_{11}P]$ which in turn is proportional to a term of the form $[A_{11}]/(1 + \gamma_1[A_{11}])$. Therefore the conservation equation takes the form $\eta_1[A_{11}] + \eta_2[A_{11}]/(1 + \gamma[A_{11}]) + \eta_3[A_{11}]/(c_1 + [A_{11}]) = A_{tot}$. Here the first term represents the combined substrates in the dephosphorylation cycle (A_{11}, A_{10}), the second term the combined effect of all complexes, and the third, the combined effect of all substrates in the phosphorylation cycle (A_{00}, A_{01}). Now the left hand side is a monotonically increasing function of A_{11} and consequently the equation can have only one (positive) root. Similarly, in the other case, $[A_{11}]$ can be written in terms of $[A_{00}]$ and an analogous type of equation can be obtained in terms of $[A_{00}]$ and the same conclusion holds

good.

Thus we have shown the absence of multistationarity in the model C1. We now show how the same approach can be used for the models C2 and C3.

Model C2. The only difference in this case is the presence of two kinases. (i) The conservation condition for kinases implies that $[K_1] = K_{1,tot}/(1 + \gamma_1[A_{00}])$ and $[K_2] = K_{2,tot}/(1 + \gamma_0[A_{01}])$. (ii) The dephosphorylation cycle is the same as that considered above, so that $[A_{10}] \propto [A_{11}]$ and both phosphatase complexes are proportional to $[A_{11}]/(1 + \gamma[A_{11}])$. (iii) By considering the steady states of $A_{00} + A_{00}K_1$ we find that $[A_{00}K_1] \propto [A_{11}P]$ and from the steady states of $A_{11} + A_{11}P$, we find that $[A_{01}K_2] \propto [A_{11}P]$. Thus $[A_{00}K_1] \propto [A_{01}K_2] \propto [A_{11}P]$. (iii) Now using the expressions for the free enzymes, we have an equation of the form $a_1[A_{00}]/(1 + \gamma_1[A_{00}]) = a_2[A_{01}]/(1 + \gamma_0[A_{01}]) = b_1[A_{11}]/(1 + \gamma[A_{11}])$, where all constants are positive. Inverting this expression, we have an equation of the form $\gamma_1/a_1 + (1/a_1)(1/[A_{00}]) = \gamma_0/a_2 + (1/a_2)(1/[A_{01}]) = \gamma_1/b_1 + (1/b_1)(1/[A_{11}])$, which is similar to the type of equation obtained above. Depending on the relative magnitudes of $\gamma_1/a_1, \gamma_0/a_2, \gamma_1/b_1$, all variables can be eliminated in favour of the variable associated with the largest of these constants. Suppose the largest of these constants is γ_1/b_1 (the other cases can be studied in exactly analogous terms as mentioned above). Then $[A_{00}] = a_{21}[A_{11}]/(1+\gamma_2[A_{11}]), [A_{01}] = a_3[A_{11}]/(1+\gamma_3[A_{11}])$. (iv) Now use the conservation condition: $[A_{00}] + [A_{00}K_1] + [A_{01}] + [A_{01}K_2] + [A_{11}] + [A_{11}P] + [A_{10}] + [A_{10}P] = A_{tot}$. All complexes are proportional to $[A_{11}P] \propto [A_{11}]/(1 + \gamma_1[A_{11}])$. So incorporating all the variable dependencies on A_{11} gives an equation of the form $\eta_1[A_{11}] + \eta_2[A_{11}]/(1 + \gamma[A_{11}]) + \eta_3[A_{11}]/(1 + \gamma_2[A_{11}]) + \eta_4[A_{11}]/(1 + \gamma_3[A_{11}]) = A_{tot}$. This represents the contributions of substrates (A_{11}, A_{10}: first term), with other terms depicting the concentration of the complexes, and the other substrates A_{00} and A_{01}. Again, we see that the form of this equation is one where the left hand side is a monotonically increasing function of A_{11}, and consequently there is only one (positive) root.

Model C3. Here there are different kinases and phosphatases, making even less of a predisposition towards bistability. (i) The conservation condition for kinases implies that $[K_1] = K_{1,tot}/(1 + \beta_1[A_{00}])$ and $[K_2] = K_{2,tot}/(1 + \beta_2[A_{01}])$. Likewise the conservation condition for the phosphatases results in $[P_1] = P_{1,tot}/(1 + \beta_3[A_{10}])$ and $[P_2] = P_{2,tot}/(1 + \beta_4[A_{11}])$. (ii) Now at steady state, by examining the steady states of different substrates and their complexes, we find that the complexes are all proportional. This means that we have an equation of the form
$a_1[A_{00}]/(1 + \gamma[A_{00}]) = a_2[A_{01}]/(1 + \gamma_0[A_{01}]) = b_1[A_{11}]/(1 + \gamma_1[A_{11}]) = b_2[A_{10}]/(1 + \gamma_2[A_{10}])$.

Taking the inverse of this equation, we have an equation of the form
$\gamma/a_1+(1/a_1)(1/[A_{00}]) = \gamma_0/a_2+(1/a_2)(1/[A_{01}]) = \gamma_1/b_1+(1/b_1)(1/[A_{11}]) = \gamma_2/b_2+(1/b_2)(1/[A_{10}])$. We then examine the largest of the constants $\gamma/a_1, \gamma_0/a_2, \gamma_1/b_1, \gamma_2/b_2$. Suppose it is γ_1/b_1: then all other substrate variables can be written in terms of the substrate variable associated with γ_1/b_1, namely A_{11} (the exact same approach can be used for other choices of the largest constant). Then
$[A_{00}] = a_2[A_{11}]/(1+\gamma_{21}[A_{11}]), [A_{01}] = a_3[A_{11}]/(1+\gamma_{31}[A_{11}]), [A_{10}] = a_4[A_{11}]/(1+\gamma_{41}[A_{11}])$. (iv)
Now use the conservation condition:
$[A_{00}] + [A_{00}K_1] + [A_{01}] + [A_{01}K_2] + [A_{11}] + [A_{11}P_2] + [A_{10}] + [A_{10}P_1] = A_{tot}$. All complexes are proportional to $[A_{11}P_2] \propto [A_{11}]/(1+\gamma_1[A_{11}])$. So incorporating all the variable dependencies on A_{11} gives an equation of the form $\eta_1[A_{11}] + \eta_2[A_{11}]/(1+\gamma_1[A_{11}]) + \eta_3[A_{11}]/(1+\gamma_{21}[A_{11}]) + \eta_4[A_{11}]/(1+\gamma_{31}[A_{11}]) + \eta_5[A_{11}]/(1+\gamma_{41}[A_{11}]) = A_{tot}$. Again, we see that the form of this equation is one where the left hand side is a monotonically increasing function of A_{11}, and consequently there is only one (positive) root.

Comment. Note that in our analysis of models C1, C2 and C3, we relied on eliminating all substrate variables in terms of one substrate variable, by (i) establishing an equation between the substrates (ii) inverting it and (iii) examining in the inverting equation, the term with the largest constant. It is possible that the largest constant may be present alongside terms for more than one variable. In this instance, the concentrations of the associated variables at steady state are proportional to one another, and the overall argument continues to hold good, since this other vairable is also a monotonically increasing function of the variable in terms of which every other variable is written.

Model C11. The main text has already shown how augmentation of the network with an extra reaction can generate bistability. However we asserted that not all augmentations can give rise to bistability. For some augmentations such as that corresponding to model $C11$, no bistability ensues. This can be easily explained. Note that the extra augmentation involves a kinase mediated conversion from A_{00} to A_{10}. Since the kinase is the same, we can describe this new reaction in two ways: one is to have A_{00} bind with K to give a distinct complex (we call it $A_{00}K_0$ dissociating to give the product. In other words we model this reaction by the equation $A_{00} + K <-- > A_{00}K_0 - > A_{10} + K$. An alternative way of describing this reaction is to have A_{00} bind to K to give $A_{00}K$, which can now give two products A_{01} and A_{10}. Both these variants yield identical results from the focal point of our analysis. (i) Exactly as in model C1, we find at steady state that $[A_{01}] \propto [A_{00}]$. It also follows from the proportionality of complexes in the phosphorylation cycle, that $[A_{00}K] \propto [A_{11}P]$, so that $[A_{00}][K] = \alpha_{12}[A_{11}][P]$ where α_{12} is a constant.

Now examining the variable A_{10} we find that $k_3[A_{11}P] + k_5[A_{00}K_0] = k_4[A_{10}P]$. Examining the steady state for $[A_{00}] + [A_{00}K] + [A_{00}K_0]$ we have $k_4[A_{10}P] = k_5[A_{00}K_0] + k_1[A_{00}K]$. Note that both the last two terms are each proportional to $[A_{00}][K]$. So this means that (i) $[A_{00}][K]$ can be written as proportional to $[A_{10}][P]$, and furthermore that $k_5[A_{00}K_0] = \alpha_0 k_4[A_{10}][P]$ where $\alpha_0 < 1$. Now looking at the equation $k_3[A_{11}P] + k_5[A_{00}K_0] = k_4[A_{10}P]$, this implies that $k_3[A_{11}P] = (1 - \alpha_0)k_4[A_{10}P]$ which then implies that $[A_{10}] \propto [A_{11}]$. Thus we see that the relationships of the different phosphoforms to each other are of an essentially similar nature as the basic cyclic model C1. (ii) This means that $P = P_{tot}/(1 + \gamma[A_{11}])$, $[K] = K_{tot}/(1 + \gamma_1[A_{00}])$ (though the constant γ_1 is different from that in model C1). The rest of the analysis is identical to that of model C1, noting that the extra complex in the substrate conservation has an identical functional form to an existing complex in that model. (iii) From the substrate conservation $[A_{00}] + [A_{00}K] + [A_{00}K_0] + [A_{01}] + [A_{01}K] + [A_{11}] + [A_{11}P] + [A_{10}] + [A_{10}P] = A_{tot}$, we see that every term can be dealt with in an identical way and the elimination of all terms in terms of one substrate variable can be done in an identical way. Again we end up with every term in the substrate conservation equation being a monotonically increasing function of the variable, again resulting in one steady state.

Other augmentations. Fig S3 presents other networks which correspond to single reaction augmentations of the basic cyclic network. (a) The common kinase common phosphatase case: here the additional models C13 and C14 are equivalent to and can be mapped on to models C11 and C12 respectively by a change of labels (for substrates and enzymes). Consequently for attractor based behaviour (as opposed to individual dose response characteristics), the behaviours of the models also map. We thus infer that model C13 does not exhibit bistability while C14 can (and can do so even with the augmented reaction in the unsaturated limit). (b) The separate kinase separate phosphatase case: here, for a similar reason, the models C33 and C34 can be mapped on to C31 and C32 respectively, to show in each case that a single augmentation in the unsaturated limit can give rise to bistability. (c) The separate kinase common phosphatase case: here the additional models C23 and C24 cannot exactly be mapped on to C21 and C22. In this regard, we make the following two points: (i) C23 can indeed exhibit bistability and even do so when the additional reaction is in the unsaturated limit (see Fig. S4). (ii) Model C24 on the other hand involves an augmentation which does not create a new complex. Thus similar to the case of model C11 analyzed above, the augmented model has the same mathematical structure as the basic cyclic network and bistability is precluded.

3 Biphasic responses.

The previous section focussed on bistability. Here we turn to biphasic responses of the fully phosphorylated form as the total concentration of a kinase is changed. Thus in contrast to the previous section we focus on a dose response curve (associated with a specific phosphoform) rather than an emergent non-linear dynamical behaviour characteristic of the overall system.

We have indicated in the main text that biphasic responses can be obtained in a number of models while they are not found in other models (in some cases, in response to specific kinase variation). We substantiate these latter conclusions analytically.

The results are organized as follows: (1) We first show that biphasic responses are ruled out in the different variants of cyclic networks with different or common kinases/phosphatases: models C1, C2 and C3. (ii) We then demonstrate the impossibility of biphasic responses in augmentations of the cyclic models: models C31, C32 and C22, when the total concentration of kinase K_1 is varied. (iii) We then show that in model C21 and C31, a robust biphasic response in response to the variation of total concentration of kinase K_2 is obtained. (iv) In the cases where biphasic responses were introduced by augmenting the basic cyclic models, we investigate if that continues to hold good if the augmented reaction operated in the unsaturated limit. The above results arise from structural considerations and are independent of kinetic parameters.

3.1 Basic Cyclic models.

We now analyze the basic cyclic models C1 , C2 and C3, using the methods of analysis employed in the context of bistability (and do not repeat those steps).

Model C1. As discussed above, at steady state $[A_{10}] \propto [A_{11}]$, $[A_{01}] \propto [A_{00}]$ and all complex concentrations are proportional. Consequently all complex concentrations are proportional to $[A_{11}P]$ (Note that here and below, when we refer to quantities being proportional, the proportionaility constants are positive and independent of total kinase concentration, for the kinase under consideration). Furthermore, using the above $[K] = K_{tot}/(1 + \gamma_1[A_{00}])$ and $P = P_{tot}/(1 + \gamma[A_{11}])$. This means that $[A_{11}P] = k_0[A_{11}]P_{tot}/(1 + \gamma[A_{11}])$. Furthermore $[A_{00}K] = k_{00}[A_{00}]K_{tot}/(1 + \gamma[A_{00}])$ and $[A_{00}K] = \beta_0[A_{11}P]$. Here $k_0, k_{00}, \beta_0, \gamma, \gamma_1$ are all positive constants (independent of the total kinase concentration).

Now suppose $d[A_{11}]/dK_{tot} = 0$, a requirement for a biphasic response. It immediately follows that $d[A_{11}P]/dK_{tot} = 0$. It also follows that $d[A_{10}]/dK_{tot} = 0$. From the proportionality of all complexes, it is

clear that the derivative of the concentration of each complex with respect to K_{tot} is zero. Now since $d[A_{00}K]/dK_{tot} = 0$, this means that $d/dK_{tot}(k_{00}[A_{00}]/(1+\gamma[A_{00}])(K_{tot})) = 0$. The right hand side is a product of two terms, one an increasing function of $[A_{00}]$ and the other K_{tot}. Differentiating and using the chain rule, we immediately see that $d[A_{00}]/dK_{tot} < 0$ and consequently $d[A_{01}]/dK_{tot} < 0$ Now from the substrate conservation condition $[A_{00}] + [A_{00}K] + [A_{01}] + [A_{01}K] + [A_{11}] + [A_{11}P] + [A_{10}] + [A_{10}P] = A_{tot}$, differentiating with respect to K_{tot} we find that all terms on the LHS have a derivative 0 (at the point of the biphasic), except for $[A_{00}]$ and $[A_{01}]$ both of which have a negative derivative. The RHS has a zero derivative, and this is a contradiction. Thus $d[A_{11}]/dK_{tot} = 0$ cannot hold good, and a biphasic response is ruled out.

Model C2. As discussed above, at steady state $[A_{11}] \propto [A_{10}]$ and all complex concentrations are proportional. Consequently all complex concentrations are proportional to $[A_{11}P]$. Furthermore, using the above $[K_1] = K_{1,tot}/(1+\beta_1[A_{00}])$ and $[K_2] = K_{2,tot}/(1+\beta_2[A_{01}])$ and $P = P_{tot}/(1+\gamma[A_{11}])$. This means that $[A_{11}P] = k_0[A_{11}]P_{tot}/(1+\gamma[A_{11}])$. Furthermore $[A_{00}K_1] = k_{00}[A_{00}]K_{1,tot}/(1+\beta_1[A_{00}])$, $[A_{01}K_2] = k_{01}[A_{01}]K_{2,tot}/(1+\beta_2[A_{01}])$ and $[A_{00}K_1] = \beta_0[A_{11}P]$. Here $k_0, k_{00}, k_{01}, \beta_1, \beta_2, \beta_0, \gamma$ are all positive constants.

As before we assume a biphasic response for the dose response curve in response to K_1 which implies the existence of a point where $d[A_{11}]/dK_{1,tot} = 0$. It immediately follows that $d[A_{11}P]/dK_{1,tot} = 0$. It also follows that $d[A_{10}]/dK_{1,tot} = 0$. Now since $[A_{01}K_2] \propto [A_{11}P]$, using the expression for $[A_{01}K_2]$ above, we see that $d[A_{01}]/dK_{1,tot} = 0$.

From the proportionality of all complexes, $d[A_{00}K_1]/dK_{1,tot} = 0$, this means that $d/dK_{1,tot}[(k_{00}[A_{00}]/(1+\gamma[A_{00}])(K_{1,tot})] = 0$. Just as before, the right hand side is a product of two terms, one an increasing function of A_{00} and the other $K_{1,tot}$. Differentiating and using the chain rule, we immediately see that $d[A_{00}]/dK_{1,tot} < 0$. Now from the substrate conservation condition $[A_{00}] + [A_{00}K_1] + [A_{01}] + [A_{01}K_2] + [A_{11}] + [A_{11}P] + [A_{10}] + [A_{10}P] = A_{tot}$, differentiating with respect to $K_{1,tot}$ we find that all terms on the LHS have a derivative 0 (at the point of the biphasic), except for $[A_{00}]$ which has a negative derivative. The RHS has a zero derivative, and this is a contradiction. Thus $d[A_{11}]/dK_{1,tot} = 0$ cannot hold good, and a biphasic response is ruled out.

Ruling out a biphasic response to variation of $K_{2,tot}$ follows in exactly similar way. The derivatives of A_{10} and all complexes with respect to $K_{2,tot}$ are zero. This means that d$[A_{00}K_1]/dK_{2,tot} = 0$ from which it follows that $d[A_{00}]/dK_{2,tot} = 0$. Since $d[A_{01}K_2]/dK_{2,tot} = 0$ it follows that $d[A_{01}]/dK_{2,tot} < 0$. This again contradicts the substrate conservation condition, since the derivative of all terms on the LHS are

zero except for $d[A_{01}]/dK_{2,tot}$ while the derivative of the RHS is zero.

Model C3. The conservation condition for kinases implies that $[K_1] = K_{1,tot}/(1+\beta_1[A_{00}])$ and $[K_2] = K_{2,tot}/(1+\beta_2[A_{01}])$. Likewise the conservation condition for the phosphatases results in $[P_1] = P_{1,tot}/(1+\beta_3[A_{10}])$ and $[P_2] = P_{2,tot}/(1+\beta_4[A_{11}])$. All complex concentrations are monotonically increasing functions of the relevant substrates $[A_{ij}E_k] = \alpha_{i,j,k}[A_{i,j}]E_{k,tot}/(1+\beta_{i,j,k}[A_{i,j}])$.

As before we assume a biphasic response for the dose response curve in response to K_1 which implies the existence of a point where $d[A_{11}]/dK_{1,tot} = 0$. It immediately follows that $d[A_{11}P_2]/dK_{1,tot} = 0$. Now since the complexes are proportional and each one of them is a monotonically increasing function of the substrate, we find that by differentiating with respect to $K_{1,tot}$, all derivatives being zero, the derivatives of the concentrations of substrates which are associated with (converted by) enzymes other that K_1 will all be zero. The only exception is $[A_{00}]$ and since $d[A_{00}K_1]/dK_{1,tot} = 0$ it follows that $d[A_{00}]/dK_{1,tot} < 0$. This now again contradicts the substrate conservation condition $[A_{00}] + [A_{00}K_1] + [A_{01}] + [A_{01}K_2] + [A_{11}] + [A_{11}P_2] + [A_{10}] + [A_{10}P_1] = A_{tot}$ since the derivative of all terms with respect $K_{1,tot}$ are zero except for one term in the LHS, while the derivative of the RHS is zero.

An exactly analogous reasoning rules out the absence of biphasic responses to $K_{2,tot}$. Exactly as before $d[A_{11}]/dK_{2,tot} = 0$ implies $d[A_{11}P_2]/dK_2tot = 0$. From the proportionality of complexes this means that the derivative of all complexes is zero, and further the derivatives of all substrates associated with an enzyme other $K2$ are zero. Since $d[A_{01}K_2]/dK_{2,tot} = 0$ it follows that $d[A_{01}]/dK_{2,tot} < 0$. This contradicts the substrate conservation condition for exactly the same reasons as mentioned above.

In conclusion biphasic responses are ruled out in model C3.

Thus far we have shown the absence of biphasic responses of $[A_{11}]$ to all relevant total kinase concentrations. Incidentally biphasic responses to all relevant total phosphatase concentrations are also ruled out for the same reasons.

3.2 Basic models with an extra reversible step.

We now examine models with an augmented step. We consider two types of augmentations: one with an extra kinase mediated reaction (mediated by K_2, in the case of multiple kinases for specificity): this is labelled by a second subscript 1 in the model (models C11, C21, C31), and the other, an extra phosphatase mediated reaction (mediated by P_2 in the case of multiple phosphatases, for specificity): this is labelled by a second subscript 2 in the model (models C12, C22, C32). In all the cases the first subscript refers to the basic cyclic model being perturbed (common kinase common phosphatase model labelled 1, different

kinase common phosphatase model labelled 2, different kinase different phosphatase model labelled 3). We first begin with models C31 and C32 (see Fig. 1) both of which can exhbit a biphasic respose to a change in total concentration of K_2. In the text we asserted that a biphasic response in response to a change in total concentration of kinase K_1 is not possible. We establish that below. At the outset, we note that the presence of an extra reaction step complicates the analysis, relative to the basic cyclic networks.

Model C31. In this model, there is an extra reaction involving enzyme K_2 converting A_{00} to A_{10}. From the conservation of enzymes, we find that $P_1 = P_{1,tot}/(1+\gamma_0[A_{10}])$ $P_2 = P_{2,tot}/(1+\gamma[A_{11}])$, $K_1 = K_{1,tot}/(1+\alpha[A_{00}])$, $K_2 = K_{2,tot}/(1+\beta_1[A_{01}]+\beta_2[A_{00}])$, where $\alpha, \beta_1, \beta_2, \gamma$ are all positive constants independent of total enzyme amounts.

Suppose there is a biphasic response as $K_{1,tot}$ is varied. (i) $d[A_{11}]/K_{1,tot} = 0$, from which it immediately follows that $d[A_{11}P_2]/K_{1,tot} = 0$. (ii) From the steady state of $A_{11} + A_{11}P_2$ we find that $[A_{01}K_2] \propto [A_{11}P_2]$. This can be written as $K_{2,tot}/(1/[A_{01}] + \beta_1 + \beta_2[A_{00}]/[A_{01}]) = \alpha_2[A_{11}P_2]$. Differentiating with respect to $K_{1,tot}$, we find the RHS is zero and so the derivative of the denominator of the LHS is zero. This means, from the chain rule that $-1/[A_{01}]^2(1+\beta_2[A_{00}])[A_{01}]' + \beta_2(1/[A_{01}])[A_{00}]' = 0$ where prime denotes derivative with respect to $K_{1,tot}$. This allows us to make two important inferences: (a) $[A_{01}]'$ and $[A_{00}]'$ have to have the same sign and also by writing the same equation as $(-1/[A_{01}]^2)[A_{01}]' + d/dK_{1,tot}([A_{00}]/[A_{01}]) = 0$, we find that the derivative of $[A_{00}]/[A_{01}]$ has the same sign as both $[A_{01}]'$ and $[A_{00}]'$ (iii) From the steady state of $A_{01} + A_{01}K_2 + A_{11} + A_{11}P_2$, we find that $[A_{00}K_1] \propto [A_{11}P_2]$. Differentiating with respect to $K_{1,tot}$, since the derivative of the LHS is zero, we conclude that $d[A_{00}]/dK_{1,tot} < 0$. From point (ii), this implies that $d[A_{01}]/dK_{1,tot} < 0$ (iv). By considering the steady state of $A_{10} + A_{10}P_1$, we find that $\alpha_3[A_{00}K_2] + \alpha_4[A_{11}P_2] = \alpha_5[A_{10}P_1]$. We differentiate this with respect to $K_{1,tot}$. The first term can be written as $\alpha_6([A_{00}]/[A_{01}])[A_{01}K_2]$. Since the derivative of $[A_{01}K_2]$ is zero (it being proportional to $[A_{11}P_2]$), from the product rule, the sign of the derivative is the sign of the derivative of $[A_{00}]/[A_{01}]$ which is negative, it being the same sign as both $[A_{01}]'$ and $[A_{00}]'$. This then means that $d[A_{10}P_1]/dK_{1,tot} < 0$. Now $[A_{10}P_1]$ is a monotonically increasing function of $[A_{10}]$ (after substituting for $[P1]$ from above), and so it immediately follows that $d[A_{10}]/dK_{1,tot} < 0$ and $d[A_{10}P_1]/dK_{1,tot} < 0$ (v) Now from the conservation condition $[A_{00}] + [A_{00}K_1] + [A_{00}K_2] + [A_{01}] + [A_{01}K_2] + [A_{11}] + [A_{11}P_2] + [A_{10}] + [A_{10}P_1] = A_{tot}$ we find that every term on the LHS has either zero or negative derivative ($[A_{11}], [A_{11}P_2], [A_{00}K_1], [A_{01}K_2]$ being the terms with zero derivative), and this leads to a contradiction. Hence the assumption of a biphasic response

is invalid.

Model C32. In this model, there is an extra reaction involving enzyme P_2 converting A_{01} to A_{00}. From the conservation of enzymes, we find that $P_1 = P_{1,tot}/(1 + \gamma_0[A_{10}])$
$P_2 = P_{2,tot}/(1 + \gamma_1[A_{11}] + \gamma_2[A_{01}])$, $K_1 = K_{1,tot}/(1 + \alpha[A_{00}])$, $K_2 = K_{2,tot}/(1 + \beta[A_{01}])$, where $\alpha, \beta, \gamma_0, \gamma_1, \gamma_2$ are all constants independent of total enzyme amounts.

Suppose there is a biphasic response as $K_{1,tot}$ is varied. (i) $d[A_{11}]/K_{1,tot} = 0$. (ii) From the steady state of $A_{11} + A_{11}P_2$, we have $[A_{01}K_2] \propto [A_{11}P_2]$. Writing everything in terms of substrate variables we have $\alpha_3[A_{01}]/(1 + \beta[A_{01}]) = \alpha_2[A_{11}]/(1 + \gamma_1[A_{11}] + \gamma_2[A_{01}])$ where $\alpha_3, \alpha_2, \gamma_1, \gamma_2$ are constants independent of $K_{1,tot}$. Rewriting this equation as
$\alpha_3[A_{01}]/(1 + \beta[A_{01}]).(1 + \gamma_1[A_{11}] + \gamma_2[A_{01}]) = \alpha_2[A_{11}]$, we differentiate with respect to $K_{1,tot}$ noting that the derivative of $[A_{11}]$ is zero. By using this fact, and the chain rule, and noting that the LHS can be written as a product of two terms which are monotonic functions of $[A_{01}]$, we obtain an equation of the form $d[A_{01}]/dK_{1,tot} = 0$. It immediately follows from the expression of K2 that $d[A_{01}K_2]/dK_{1,tot} = 0$ (iii) The above fact then means that (a)$d[P_2]/dK_{1,tot} = 0$, $d[A_{11}P_2]/dK_{1,tot} = 0$,$d[A_{01}P_2]/dK_{1,tot} = 0$ (iv) Now we focus on $[A_{10}]$. Since $[A_{10}P_1] \propto [A_{11}P_2]$ and since $[P_1]$ depends only on $[A_{10}]$ as seen above, it immediately follows that $d[A_{10}]/dK_{1,tot} = 0$ and $d[A_{10}P_1]/dK_{1,tot} = 0$ (v) Now we focus on the steady state of $A_{00} + A_{00}K_1$. This results in an equation of the form $\alpha_5[A_{00}K_1] = \alpha_6[A_{10}P_1] + \alpha_7[A_{01}P_2]$, where $\alpha_5, \alpha_6, \alpha_7$ are all independent of $K_{1,tot}$. Now differentiating this equation, we find that $d[A_{00}K_1]/dK_{1,tot} = 0$, and by substituting for K_1 from above, we find $d[A_{00}]/dK_{1,tot} < 0$. (vi) From the substrate conservation condition we have
$[A_{00}] + [A_{00}K_1] + [A_{01}] + [A_{01}K_2] + [A_{01}P_2] + [A_{11}] + [A_{11}P_2] + [A_{10}] + [A_{10}P_1] = A_{tot}$, and differentiang we obtain a contradiction as all terms on the LHS have a zero derivative except for the first term which is negative. This invalidates the original assumption.

Model C22. We now show that this model is also incapable of exhibiting a biphasic dose response curve as the total amount of K_1 is varied. In many respects this is similar to the case of model 32. We first note from the conservation conditions for enzymes $P = P_{tot}/(1 + \gamma_1[A_{11}] + \gamma_2[A_{10}] + \gamma_3[A_{01}])$,
$K_1 = K_{1,tot}/(1 + \alpha[A_{00}])$, $K_2 = K_{2,tot}/(1 + \beta[A_{01}])$, where $\alpha, \beta, \gamma_1, \gamma_2, \gamma_3$ are all constants independent of total enzyme amounts. From the steady state of $A_{10} + A_{10}P$, we find that $[A_{10}] \propto [A_{11}]$.

Suppose $d[A_{11}]/dK_{1,tot} = 0$. This straightaway implies that $d[A_{10}]/dK_{1,tot} = 0$ (ii) From the steady state of $A_{11} + A_{11}P$, we have $[A_{01}K_2] \propto [A_{11}P]$. By writing the expression for P and eliminating A_{10} we have an equation of the form $\alpha_1[A_{01}]K_{2,tot}/(1 + \beta[A_{01}]) = \alpha_2[A_{11}]/(1 + \gamma_4[A_{11}] + \gamma_5[A_{01}])$.

This can be rewritten as $\alpha_1[A_{01}]K_{2,tot}/(1+\beta[A_{01}]).(1+\gamma_4[A_{11}]+\gamma_5[A_{01}]) = \alpha_2[A_{11}]$. Now differentiate with respect to $K_{1,tot}$. The LHS is a product of two functions which are monotonically increasing functions of $[A_{01}]$. The derivative of the RHS is zero. Differentiating, we find an equation of the form $F([A_{01}],[A_{11}])d[A_{01}]/dK_{1,tot} = 0$,where $F > 0$, from which it follows that $d[A_{01}]/dK_{1,tot} = 0$ (iii) It immediately follows that
$dP/K_{1,tot} = 0, d[A_{11}P]/dK_{1,tot} = 0, d[[A_{10}P]/dK_{1,tot} = 0, d[[A_{01}P]/dK_{1,tot} = 0$. This also leads to the fact that $d[A_{01}K_2]/dK_{1,tot} = 0$ (iv) Now from the steady state of $A_{00} + A_{00}K_1$, we have an equation of the form $\alpha_3[A_{00}K1] = \alpha_4[A_{01}P] + \alpha_5[A_{10}P]$. Differentiating, we find that the derivative of the RHS with respect to $K_{1,tot}$ is zero, implying the same for $d[A_{00}K_1]/dK_{1,tot}$. By expressing the dependence of K_1 on $[A_{00}]$, we again find $d[A_{00}]/dK_{1,tot} < 0$. Now in the conservation condition
$[A_{00}] + [A_{00}K_1] + [A_{01}] + [A_{01}K_2] + [A_{01}P] + [A_{11}] + [A_{11}P] + [A_{10}] + [A_{10}P] = A_{tot}$, we find that all terms on the LHS have a zero derivative except for the first term, and the RHS has zero derivative. This results in a contradiction. Thus a biphasic response of the type assumed can be ruled out.

Model C11. We have already analyzed model C11 in the context of bistability. We have shown that the extra reaction introduces an extra complex of a preexisting type. Thus the behaviour of this model is very similar to that of model C1, and by eliminating variables, we see the same types of dependencies between variables, with the only alterations being in the constants. For an exactly analogous reason, biphasic responses can be ruled out, exactly as they were in model C1. We will consequently not repeat what is essentially an identically parallel analysis.

Model C21. Model C21 exhibits a robust biphasic response with regard to the variation of the total concentration of K_2. This is seen for all values of parameters. The reason for this can be seen as follows. When K_2 is absent $[A_{11}] = 0$ simply because there is no reaction producing it. As K_2 increases $[A_{11}]$ increases. Interestingly as $K_{2,tot} \to \infty$, $[A_{11}] \to 0$. This can be seen intuitively from the network C21 itself. For very large $K_{2,tot}$, most of the reaction (involving the conversion of A_{00}) involves the conversion of A_{00} to A_{10}, with negligible flux to the A_{01} reaction: in fact in relative terms the flux to this pathway approaches 0. Consequently, $[A_{11}]$ approaches zero as it is dependent on this reaction, not being produced by any other pathway. This then implies that there necessarily has to be a biphasic response (or a multiphasic response), and monotonic dose response curves are precluded.

Model C31. Model C31 shares a similar structure as model C21 (with respect to enzyme K_2). Consequently, a biphasic response to the variation of K_2 total concentration is guaranteed. Here too, we see that when $K_{2,tot} = 0$, $[A_{11}] = 0$, while $K_{2,tot} \to \infty$, $[A_{11}] \to 0$. The reason for this is exactly the same

as above. For large values of $K_{2,tot}$, the conversion of A_{00} is predominantly to A_{10} with negligible flux in the upper branch of reactions, proceeding through A_{01} (Fig. 1). Thus, there necessarily has to be a biphasic (or multiphasic response) and monotonic dose-response curves are precluded.

3.2.1 The role of enzyme sequestration in generating biphasic responses.

Our analysis above has primarily focussed on ruling out biphasic responses in a number of augmentations of the basic cyclic models. Simulations, on the other hand do show biphasic responses for all the cases summarized in Table 1. This includes models C21, C22, C31, C32 in response to total concentration of K_2, Model C21 in response to total concentration of K_1, and model C12 in response to total concentration of K. Each of these models has one extra reaction which enables this biphasic response (noting that the basic cyclic models do not exhibit the biphasic response). However, the question which remains is whether a biphasic response persists even if there is no enzyme sequestration in this extra reaction, i.e. if this extra reaction operates in an unsaturated regime.

We justify, below the answer to this: (i) With respect to variation of concentration of K_2, models C21 and C31 exhibit a biphasic response even without enzyme sequestration, while models C22 and C32 do not. (ii) Model C12 does not exhibit a biphasic response to K, if the additional reaction is not associated with sequestration.

We make a comment with regard to our analysis below. The extra reaction acting in an unsaturated regime implies that there is negligible sequestration of enzyme and negligible complex. In our analysis the negligible sequestration of the enzyme emerges as a key factor in the analysis. We note that while the relevant complex concentration is low, the associated reaction rate in general is not. In the analysis, we retain the complex as in the analysis above everywhere, except in the enzyme conservation equation, and draw all our conclusions from this.

A slightly different way of doing this is to eliminate the relevant complex in both enzyme and substrate conservation equations, and using the associated rate of the reaction (where it is used) as proportional to the product of the relevant free enzyme and substrate. This analysis is essentially similar to our analysis, since all our analysis requires about the complex (apart from the negligible sequestration of enzyme therein) is the fact that the concentration of the complex is proportional to that of the free enzyme and substrate, anyway and (ii) the presence of the complex in the substrate conservation does not alter the argument in any essential way. In its absence there is one less term to deal with, but the essential argument deployed works in an identical way.

Models C21 and C31. As discussed above, both these models result in robust biphasic responses, and as $K_{2,tot} \to \infty$, $[A_{11}] \to 0$. This does not in any way depend on K_2 being sequestered in the additional reaction, and purely depends on how K_2 appears in the reaction network. Consequently biphasic responses can be obtained without sequestration of enzyme in the extra reaction.

Model C22. From the conservation of enzymes, we have: $P = P_{tot}/(1 + \gamma_1[A_{11}] + \gamma_2[A_{10}])$, $K_1 = K_{1,tot}/(1 + \alpha[A_{00}])$, $K_2 = K_{2,tot}/(1 + \beta[A_{01}])$, where $\alpha, \gamma_1, \gamma_2$ are all constants independent of total enzyme amounts. From the steady state of $A_{10} + A_{10}P$, we find that $[A_{10}] \propto [A_{11}]$. The absence of the effect of $[A_{01}]$ in the free phosphatase concentration simplifies matters: Suppose $d[A_{11}]/dK_{2,tot} = 0$, it immediately follows that the following terms also have zero derivatives with respect to $K_{2,tot}$: $[A_{10}], [A_{11}P], [A_{10}P], [P]$. Further, since at steady state $[A_{01}K_2] \propto [A_{11}P]$, it also follows that $[A_{01}K_2]$ also has zero derivative as well, implying that the derivative of $[A_{01}]$ is negative. The remaining steps are similar to those above. From the steady state of $A_{00} + A_{00}K_1$, we have an equation of the form $\alpha_3[A_{00}K_1] = \alpha_4[A_{01}][P] + \alpha_5[A_{10}P]$. Differentiating with respect to $K_{2,tot}$, we find that the derivative of the RHS with respect to $K_{2,tot}$ is negative, (since the derivative of $[A_{01}] < 0$ from above, and the derivative of $[P]$ is zero, implying the same for $d[A_{00}K_1]/dK_{2,tot}$ and consequently (using the expression for K_1 above) $d[A_{00}]/dK_{2,tot} < 0$. Now in the conservation condition $[A_{00}] + [A_{00}K_1] + [A_{01}] + [A_{01}K_2] + [A_{01}P] + [A_{11}] + [A_{11}P] + [A_{10}] + [A_{10}P] = A_{tot}$, we find that all terms on the LHS have a zero derivative except for $[A_{01}], [A_{00}], [A_{00}K_1]$ (and $[A_{01}P]$ if not neglected) terms, and the RHS has zero derivative. This results in a contradiction. Thus a biphasic response of the type assumed can be ruled out.

Model C32. From the conservation of enzymes, we have: $P_1 = P_{1,tot}/(1 + \gamma_0[A_{10}])$ $P_2 = P_{2,tot}/(1 + \gamma_1[A_{11}])$, $K_1 = K_{1,tot}/(1 + \alpha[A_{00}])$, $K_2 = K_{2,tot}/(1 + \beta[A_{01}])$, where $\alpha, \beta, \gamma_0, \gamma_1$ are all constants independent of total enzyme amounts. Suppose $d[A_{11}]/dK_{2,tot} = 0$, it immediately follows that the following terms also have zero derivatives $[A_{10}], [A_{11}P_2], [A_{10}P_1]$ (this follows from the proportionality of the concentrations of complexes $[A_{11}P_2]$ and $[A_{10}P_1]$). Further, since at steady state $[A_{01}K_2] \propto [A_{11}P]$, it also follows that both $[A_{01}K_2]$ also has zero derivative, while $[A_{01}]$ has a negative derivative. The remaining steps are similar to the analysis of model C22 above. Now we focus on the steady state of $A_{00} + A_{00}K_1$. This results in an equation of the form $\alpha_5[A_{00}K_1] = \alpha_6[A_{10}P_1] + \alpha_7[A_{01}][P_2]$, where $\alpha_5, \alpha_6, \alpha_7$ are all independent of $K_{2,tot}$. Now differentiating this equation, we find that $d[A_{00}K_1]/dK_{2,tot} < 0$, and consequently, we find $d[A_{00}]/dK_{2,tot} < 0$ (this is due the negative derivative of $[A_{01}]$ and zero derivative of P_2). (vi) From the substrate conservation condition we have

$[A_{00}] + [A_{00}K_1] + [A_{01}] + [A_{01}K_2] + [A_{01}P_2] + [A_{11}] + [A_{11}P_2] + [A_{10}] + [A_{10}P_1] = A_{tot}$, and differentiating we obtain a contradiction as all terms on the LHS have a zero derivative except for $[A_{01}], [A_{00}], [A_{00}K_1]$ (and $[A_{01}P_2]$ if it is not neglected) which have negative derivatives. This invalidates the original assumption.

Model C12.. From the conservation of enzymes, we have $[P] = P_{tot}/(1 + \gamma_1[A_{11}] + \gamma_2[A_{10}])$, $[K] = K_{tot}/(1 + \alpha_1[A_{00}] + \alpha_2[A_{01}])$. Furthermore at steady state $[A_{10}] \propto [A_{11}]$. Now suppose $d[A_{11}]/dK_{tot} = 0$, it follows that the following quantities also have zero derivatives: $[A_{11}P], [A_{10}], [A_{10}P]$. Furthermore since $[A_{01}K] \propto [A_{11}P]$ it follows that $[A_{01}K]$ has a zero derivative.

We need to assess the remaining 4 terms $[A_{00}], [A_{00}K], [A_{01}P], [A_{01}]$. This needs a simultaneous consideration of all these factors, and their interrelationships. To do this, we consider two basic cases: (i) Case 1: Suppose $d[A_{01}]/dK_{tot} > 0$. Then it immediately follows that $d[A_{01}P]/dK_{tot} > 0$. Furthermore from the steady state of $A_{01} + A_{01}K + A_{01}P$, we have a relationship between the complexes of the form $\alpha_3[A_{01}][P] + \alpha_4[A_{01}K] = \alpha_5[A_{00}K]$. Differentiating with respect to K_{tot}, the first term has a positive derivative, while the second one has zero derivative, so it immediately follows that $d[A_{00}K]/dK_{tot} > 0$. It only remains to assess $[A_{00}]$. To do this we use $d[A_{01}K]/dK_{tot} = 0$

Using the expression for K, we find that implies $d/dK_{tot}(\alpha_6[A_{01}]/(1 + \alpha_1[A_{00}] + \alpha_2[A_{01}])][K_{tot}]) = 0$ which implies that $d/dK_{tot}[\alpha_6[A_{01}]/(1 + \alpha_1[A_{00}] + \alpha_2[A_{01}])] < 0$. Moving all variables to the denominator, this implies that the derivative of the denominator is positive, i.e. $d/dK_{tot}(1/[A_{01}] + \alpha_1[A_{00}]/[A_{01}]) > 0$. Differentiating this, we immediately see that $d[A_{00}]/dK_{tot} > 0$. This now contradicts the conservation condition for substrate: $[A_{00}] + [A_{00}K] + [A_{01}] + [A_{01}K] + [A_{11}] + [A_{11}P] + [A_{10}] + [A_{10}P] = A_{tot}$, since four of the derivatives have the same sign while the other five have zero derivative.

(ii) Case 2: $d[A_{01}]/dK_{tot} < 0$. It immediately follows that $d[A_{01}P]/dK_{tot} < 0$, and again just as above, from the equation $\alpha_3[A_{01}][P] + \alpha_4[A_{01}K] = \alpha_5[A_{00}K]$, it follows that $d[A_{00}K]/dK_{tot} < 0$. We use this to infer the sign of the derivative $[A_{00}]$. It follows from the experssion for $[A_{00}K]$ that $d/dK_{tot}(\alpha_7[A_{00}]/(1 + \alpha_1[A_{00}] + \alpha_2[A_{01}])][K_{tot}]) < 0$, which implies that $d/dK_{tot}\alpha_7[A_{00}]/(1 + \alpha_1[A_{00}] + \alpha_2[A_{01}]) < 0$. Again, just as above, taking all the variables to the denominator, we have $d/dK_{tot}(1/[A_{00}] + \alpha_2[A_{01}]/[A_{00}]) > 0$. Differentiating term by term, we see that this necessarily implies that $d[A_{00}]/dK_{tot} < 0$. Again this contradicts the substrate conservation equation because the four terms have derivatives of the same signs (negative), while the other five have zero derivative

(iii) Case 3: We show that the possibility of $d[A_{01}]/dK_{tot} = 0$ can be ruled out.If this were the case, this means that $d/dK_{tot}[A_{01}][P] = d/dK_{tot}[A_{00}K] = 0$. Thus $d[A_{00}K]/dK_{tot} = 0$. However the fact that $d/dK_{tot}[A_{01}K] = 0$ implies $dK/dK_{tot} = 0$ which when combined with a zero derivative for $[A_{00}K]$implies $d[A_{00}]/dK_{tot} = 0$. But this contradicts $dK/dK_{tot} = 0$. So this scenario does not occur.

All in all we rule out biphasic behaviour in model C12, if the extra reaction does not involve enzyme sequestration.

Biphasic response in model C21. We have already showed that the biphasic response in model C21 to variation of K_2 does not need sequestration of enzyme in the additional step. We now demonstrate that this is also true for the enzyme K_1. The reasoning here is different, and we note that the biphasic response to K_1 is not as widespread as the biphasic response to K_2. Our approach here is to analyze a tractable case to demonstrate the origins of this biphasic behaviour in model C21. We make the following assumptions: (i) K_2 acts in the unsaturated limit, as does K_1. (ii) The dephosphorylation of A_{11} also occurs in the unsaturated limit.

This means that the conservation conditions result in $[K_1] = K_{1,tot}$, $[K_2] = K_{2,tot}$ and $[P] = P_{tot}/(1 + \alpha_1[A_{10}])$. From the various steady state equations we have $[A_{01}][K_2] = \alpha_2[A_{11}][P], [A_{00}][K1] = \alpha_3[A_{11}][P]$. Furthermore, $\alpha_4[A_{10}][P] = \alpha_5[A_{11}][P] + \alpha_6[A_{00}][K_2] = (\alpha_5 + \alpha_6/\alpha_3[K_2]/[K_1])[A_{11}][P]$, which implies $[A_{10}] = (1/\alpha_4)(\alpha_5 + (\alpha_6/\alpha_3)([K_2]/[K_1]))[A_{11}] = (\gamma_1 + \gamma_2[K_2]/[K_1])[A_{11}]$. Thus all the variables can be written in terms of $[A_{11}]$. Furthermore $[A_{10}P] = \alpha_7(\gamma_1 + \gamma_2[K_2]/[K_1])[A_{11}]][P]$.

By employing the conservation condition for substrate we have the equation $[A_{11}](1 + (\gamma_1 + \gamma_2[K_2]/[K_1])) + P(\alpha_3/[K_1] + \alpha_2/[K_2]) + \alpha_7(\gamma_1 + \gamma_2[K_2]/[K_1])P = A_{tot}$. Further $P = P_{tot}/(1 + \alpha_1[A_{10}]) = P_{tot}/(1 + \alpha_1(\gamma_1 + \gamma_2[K_2]/[K_1])[A_{11}])$.

Firstly we note here that if there were no phosphatase sequestration at all i.e. $\alpha_7 = 0$ it is easy to see that even here as $[K_2] \to \infty, [A_{11}] \to 0$. This further substantiates the fact that a biphasic response in $[K_2]$ can be obtained without any sequestration effects in the additional reaction, and we see this to be the case in an even simpler setting where there is no sequestration of any kind.

We make one assumption on parameters to explicitly reveal the origins for biphasic response, more transparently. We assume that term the denominator of the expression for P, we can neglect 1 in relation to $\alpha_1(\gamma_1 + \gamma_2[K_2]/[K_1])[A_{11}])$. Then $P \approx P_{tot}/(\alpha_1(\gamma_1 + \gamma_2[K_2]/[K_1])[A_{11}])$. This then allows us to write the expression for $[A_{11}]$ as $[A_{11}] =$ $(1/[1+\gamma_1+\gamma_2[K_2]/[K_1]])(A_{tot} - [\alpha_3/[K_1] + \alpha_2/[K_2] + \alpha_7(\gamma_1 + \gamma_2[K_2]/[K_1])]/\alpha_1(\gamma_1 + \gamma_2[K_2]/[K_1])$.

This can be simplified as

$[A_{11}] = (1/[1+\gamma_1+\gamma_2[K_2]/[K_1]])(A_{tot} - \alpha_7\gamma_1/\alpha_1 - [\alpha_3/[K_1]+\alpha_2/[K_2]]/(\alpha_1(\gamma_1+\gamma_2[K_2]/[K_1])))$.

If we neglect the last group of terms, then no biphasic response to $[K_1]$ can be obtained. However this last grouping of terms demonstrates the ingredients for a tradeoff. Examining the term $[\alpha_3/[K_1]+\alpha_2/[K_2]]/(\alpha_1(\gamma_1+\gamma_2[K_2]/[K_1])))$, we see that this can exhibit both an increasing and a decreasing dependence on $[K_1]$ depending on parameters (similar to analysis of functions of the type $(ax+b)/(cx+d)$. In particular, if this exhibits an increasing dependence on $[K_1]$, then we see the origins of a tradeoff in the $[K_1]$ response: the denominator decreases, but the overall numerator in the LHS also decreases. Indeed simulations readily show that a biphasic response may be obtained.

4 Parameter values in Figures

We present here some details regarding the parameters employed in the computational results presented in the main text. The parameters are all dimensionless parameters. The paper analyses the information processing capabilities of cyclic multi-site phosphorylation systems to make focussed conclusions regarding the possibility of different types of behavior. In all the numerical simulation and bifurcation analysis, unless otherwise mentioned, the initial conditions of substrate, also equal to total concentration of substrate (A_{tot}), and phosphatase, (equal to total phosphatase, P_{tot}), are 40 and 1, respectively. While the initial condition for the substrate corresponds to the substrate being present in the unmodified form, the initial condition for the kinase and phosphatase is for all the enzyme which is in the active form. In any case the concentrations of the various complexes are zero initially. Furthermore, kinetic parameters such as binding, unbinding and catalytic constants are equal to 1, unless otherwise mentioned below.

Figure 3 (a) Distributive Model (Model S_1) or Model C_1: k_1 = 100 and k_3 = 0.01 (b) Distributive Model (Model S_1) or Model C_1: k_6 = 0.01 and k_9 = 5

Figure 4 (a) Model S_1: k_4 = 0.1, k_6 = 4, k_7 = 50, k_{12} = 2 (b) Model C_1: (i) k_4 = 0.1, k_6 = 4, k_7 = 50 and k_{12} = 2 (ii) k_4 = 0.1, k_6 = 4, k_7 = 50, k_{12} = 2 and K_{tot} = 1.3 (c) Model C2: k4 = 0.1, k6 = 4 and k7 = k12 = 50 (i) $K_{2,tot}$ = 4 (ii) $K_{2,tot}$ = 4.5 (iii) $K_{2,tot}$ = 5.5

Figure 5 (a) Model C_{12}: k_4 = 0.1, k_6 = 4, k_{12} = 2, k_{13} = 200 and k_{15} = 40 (b) Modified model C_2 (i) Model C_{21}: k_6 = 9, k_7 = k_9 = k_{10} = 100, k_{13} = 200, k_{15} = 40 and $K_{1,tot}$ = 5 (ii) Model C_{22}: k_1 = k_{13} = k_{15} = 100 (c) Modified model C3 (i) Model C_{31}: k_{13} = 200, k_{15} = 40 and $K_{2,tot}$ = 0.1 (ii) Model C_{32}: k_1 = 100, k_3 = k_9 = 0.01 and $K_{2,tot}$ = 0.1

Figure 6 (a) Model C_{31}: k_1 = 100, k_4 = 2.5, k_6 = 100, k_{10} = 0.1, k_{12} = k_{13} = 100, $P_{1,tot}$ = $P_{2,tot}$ = 1.5 and $K_{2,tot}$ = 0.5 (b) Modified model C_2 (i) Model C_{22}: k_1 = 100, k_4 = 0.1, k_6 = 4, k_7 = 100, k_{10} = 2.5, k_{12} = 180, k_{13} = 0.5, k_{15} = 5 and $K_{2,tot}$ = 1.5 (ii) Model C_{21}: k_4 = 0.1, k_6 = 4, k_7 = 6, k_{10} = 2.5, k_{12} = 200, k_{13} = k_{14} = k_{15} = 0.1 and $K_{1,tot}$ = 1.5

Supplementary Figures. All parameter values unless stated otherwise are 1. Total concentration for substrates and enzymes unless otherwise stated are 5.

The Supplementary Fig. S2 deals with the behaviour of cyclic models with an augmented reaction in the unsaturated limit. Figure S_2

a. Model C_{12}: k_1 = 10, k_4 = 10, k_6 = 5, k_7 = 5, k_9 = 10, k_{10} = 10, k_{12} = 10, k_{13} = 2, A_{tot} = 25,P_{tot}= 2;

b. Model C_{21}: k_3 = 10, k_4 = 10, k_9 = 5, k_{10} = 2, k_{12} = 2, k_{13} = 5, A_{tot} = 50;

Model C_{22}: k_3 = 1.15, k_4 = 2, k_6 = 5, k_7 = 6, k_{12} = 4, k_{13} = 5, A_{tot} = 50;

c. Model C_{31}: k_4 = 10, k_7 = 2, k_9 = 1.5, k_{10} = 5, k_{12} = 5, k_{13} = 5, A_{tot} = 150;

Model C_{32}: k_1 = 5, k_3 = 5, k_7 = 10, k_{10} = 2, k_{12} = 1.5, k_{13} = 5, A_{tot} = 150;

d. Model C_{31}:k_3 = 10; k_4 = 10, k_6 = 2, k_7 = 2, k_9 = 1.5, k_{10} = 5, k_{12} = 5, k_{13} = 5, A_{tot} = 150;

Supplementary Fig. S4 A. s_1 = 3; s_{b1} = 1; s_{ub1} = 1; k_1 = 10; k_2 = 1; k_3 = 5; k_4 = 2; A_{Total} = 100; $K2_{Total}$ = 2; P_{Total} = 4; B. s = 0.5; k_1 = 1; k_2 = 4; k_3 = 3; k_4 = 2; A_{Total} = 100; $K2_{Total}$ = 2; P_{Total} = 4;

Supplementary figure captions.

Supplementary Figure 1: Depiction of the reaction network in cyclic, distributive double-site phosphorylation models. Schematic reaction models of double-site cyclic phosphorylation with multiple variants of mechanisms involving either common or different kinases or phosphatases are depicted. (a) presents the sequential distributive phosphorylation model as a reference whereas (b-d) presents double-site cyclic phosphorylation with multiple variants of mechanisms involving either common or different kinases or phosphatases. (e) presents enzymatic reaction augmented in modified cyclic models.

Supplementary Figure 2: (A-C). Bistability in the modified cyclic models with an augmentation, where the augmented reaction operates in the unsaturated limit (D). Presence of both multi-stationarity and oscillations in model C_{31}. This demonstrates that the sequestration of an enzyme in the complex (in the augmented reaction) is not a prerequisite for inducing bistability or oscillations in these models, through an augmented reaction. Connected lines indicate stable steady states, while dotted lines indicate unstable steady states. LP, HP represent a limit point and a Hopf bifurcation, respectively. Dark blue lines in (D)

indicate bounds on concentration in the oscillatory branch emerging out of the Hopf bifurcation.

Supplementary Figure 3: Schematic representation of the various configurations of modified cyclic double site modifications involving an additional enzymatic reaction.

Supplementary Figure 4: Bistability in the modified cyclic model with an augmentation (C23). (A). In the full model (C23). (B). When the augmented reaction operates in the unsaturated limit. [Dashed and thick lines represent unstable and stable steady states respectively; LP and H denote a limit point and Hopf bifurcation point respectively].
